# Highly accurate and large-scale collision cross sections prediction with graph neural networks

**DOI:** 10.1038/s42004-023-00939-w

**Published:** 2023-07-04

**Authors:** Renfeng Guo, Youjia Zhang, Yuxuan Liao, Qiong Yang, Ting Xie, Xiaqiong Fan, Zhonglong Lin, Yi Chen, Hongmei Lu, Zhimin Zhang

**Affiliations:** 1 College of Chemistry and Chemical Engineering, Central South University, 410083 Changsha, China; 2 School of Computer Science and Technology, Huazhong University of Science and Technology, 430074 Wuhan, China; 3 Yunnan Academy of Tobacco Agricultural Sciences, 650021 Kunming, Yunnan China

**Keywords:** Cheminformatics, Mass spectrometry, Metabolomics

## Abstract

The collision cross section (CCS) values derived from ion mobility spectrometry can be used to improve the accuracy of compound identification. Here, we have developed the Structure included graph merging with adduct method for CCS prediction (SigmaCCS) based on graph neural networks using 3D conformers as inputs. A model was trained, evaluated, and tested with >5,000 experimental CCS values. It achieved a coefficient of determination of 0.9945 and a median relative error of 1.1751% on the test set. The model-agnostic interpretation method and the visualization of the learned representations were used to investigate the chemical rationality of SigmaCCS. An in-silico database with 282 million CCS values was generated for three different adduct types of 94 million compounds. Its source code is publicly available at https://github.com/zmzhang/SigmaCCS. Altogether, SigmaCCS is an accurate, rational, and off-the-shelf method to directly predict CCS values from molecular structures.

## Abstract (web summary)

The collision cross section (CCS) values derived from ion mobility spectrometry (IMS) are a significant molecular property used for compound identification, however, accurate CCS prediction from molecular structure remains challenging. Here, the authors develop an accurate SigmaCCS approach based on graph neural networks using 3D conformers generated from SMILES strings to directly predict CCS values from molecular structures.

## Introduction

Liquid chromatography-mass spectrometry (LC-MS) is an ideal platform for analyzing complex samples^1^. The computational pipeline from raw LC-MS data to discriminant models can be easily constructed with existing packages, such as XCMS^2^ and MetaboAnalyst^3^. Furthermore, the potential biomarkers (compounds) can be screened with assistance from these models. However, only a limited percentage of compounds can be identified by searching MS/MS spectral databases due to the limited molecular coverage of databases and the unavailability of pure standards^4^. To partially address this problem, researchers have taken advantage of structural databases and computational methods to generate mass spectra from molecular structures with rules or models^5,6^, predict molecular fingerprints from mass spectra^7^, extend the identifiable molecular space using spectrum-structure similarity^8,9^, and simulate mass spectra with quantum chemical calculation^10^. However, compound identification remains a major challenge when analyzing complex samples. Therefore, it is necessary to use other stable, distinguishable, and readily obtained physicochemical properties to improve the performance of compound identification^11^.

Recently, ion mobility spectrometry (IMS) has been integrated into LC-MS^12^. The collision cross section (CCS) values can be calculated from the drift time measured by IMS. The CCS values have excellent reproducibility^13^ and are highly correlated with the molecular shapes^14^. It provides an additional physicochemical property that can be used to improve identification accuracy^15^. However, this utility is limited by the availability of reference CCS values. The theoretical calculation and model-based prediction methods have been developed to obtain CCS values from molecular structures. The common theoretical calculation methods include projection approximation, exact hard-spheres scattering, and trajectory method (TM), which have been implemented in the MobCal program^16^ and in silico chemical library engine (ISiCLE)^17^. Nevertheless, the projection approximation and exact hard-sphere scattering methods are not accurate enough, and the TM method is time-consuming for large-scale molecules. The model-based prediction methods use machine learning to establish the relationship between the molecular structures and their experimental CCS values. They have been used with molecular descriptors to predict CCS values (or related properties such as reduced ion mobility constant and drift time). Those methods involve multiple linear regression^18^, random forests^19^, partial least squares^20^, artificial neural network^21^, and support vector regression^22–24^. The issue with these methods is that the descriptors do not always completely reflect the structural features of compounds. They may lose some useful information for CCS prediction.

Deep learning methods can take simplified molecular-input line entry system (SMILES) strings or molecular graphs as inputs and learn multilevel representations from chemical datasets to predict molecular properties. They have achieved state-of-the-art performance in related fields ranging from peak detection^25^, alignment^26^, retention time (RT)^27–29^ and CCS prediction^30,31^ to library searching^32^ and in silico compound identification^33–35^. For instance, DeepCCS can predict the CCS values of molecules with a convolutional neural network (CNN) from the one-hot encoding of their SMILES strings, and it has achieved good performance with the coefficient of determination (R^2^) and median relative error (Median RE) of 0.976 and 2.67%, respectively^30^. According to the theoretical calculation methods, the CCS value of a molecule is closely related to its 3D structure, which is not included in the one-hot encoding of its SMILES string. Therefore, there is plenty of room to improve the accuracy of CCS prediction with proper neural network architectures and reasonable inputs.

**Fig. 1:**
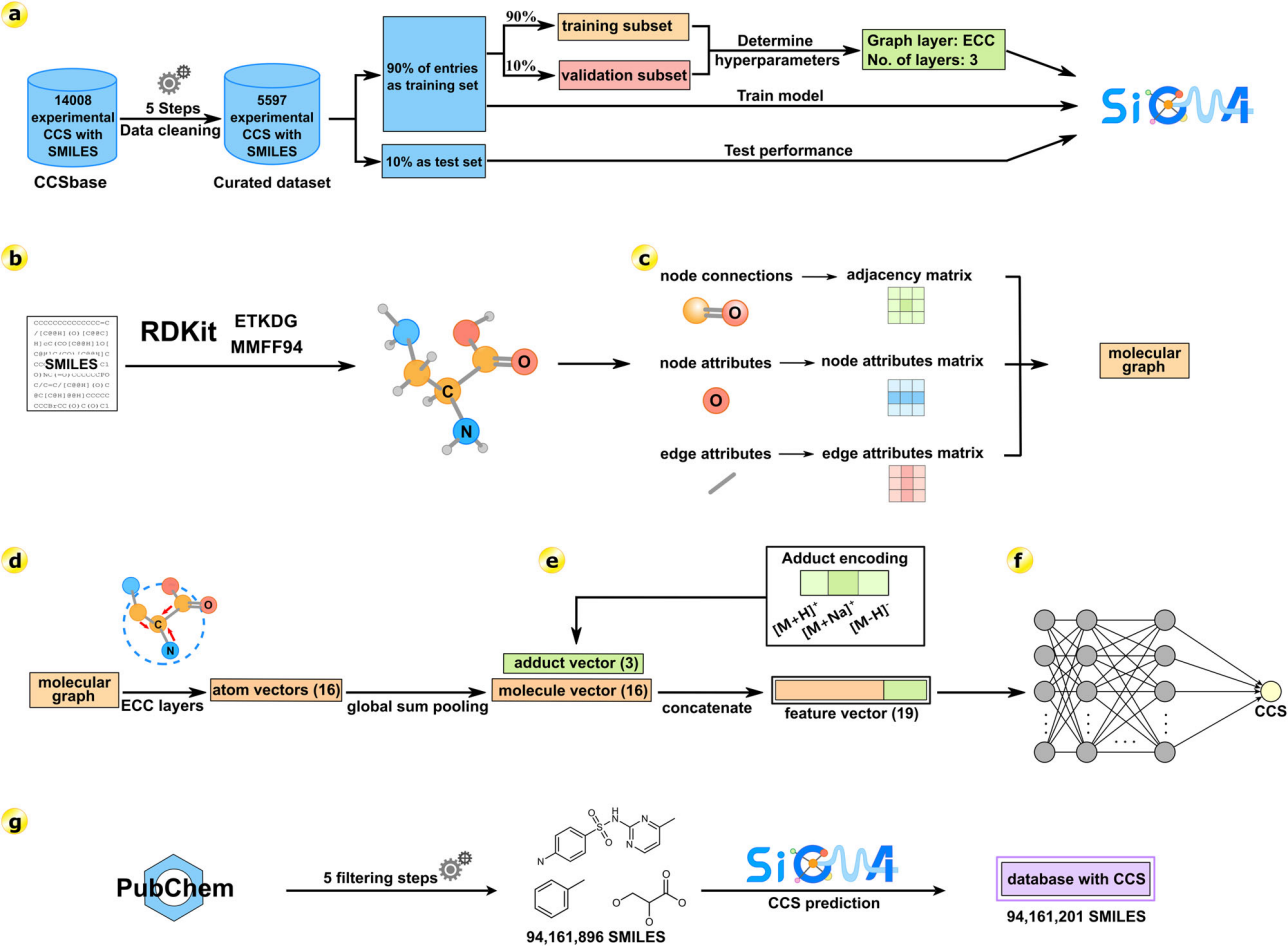
Workflow of SigmaCCS. **a** Dataset curation: a curated dataset with 5597 experimental CCS values was used to train, validate and test the SigmaCCS model. It was obtained through a five-step cleaning pipeline from CCSbase. **b** Conformer generation: the molecular object of each molecule was constructed from its SMILES string, and the 3D conformer was generated and optimized by ETKDG and MMFF94. The attributes of each atom and bond in the molecule were calculated by RDKit. **c** Molecular graph construction: the molecular graph of each molecule was established by initializing the node attribute matrix, the edge attribute matrix, and the adjacency matrix with attributes calculated in the previous step and its connection table. **d** Edge-conditioned convolution: the atomic vector of each atom in the molecule was learned from the curated dataset with edge-conditioned convolution, and the molecular vector was generated from atom vectors through global sum pooling. **e** Adduct encoding: the adduct ion type ([M + H]^+^, [M+Na]^+^, and [M-H]^-^) was encoded as a one-hot vector. The molecular vector and the one-hot vector of adduct type were concatenated to obtain the feature vector. **f** CCS prediction: the feature vector was fed into the fully connected layers and feedforwarded to the output layer to predict the CCS value. **g** Database generation: the SigmaCCS model was used to predict CCS values of 94,161,201 compounds in PubChem. Three different adduct ions of each molecule were predicted. There are >280,000,000 predicted CCS values in the CCS database. The complete workflow of SigmaCCS was implemented in Python (v3.7.7).

In this study, we developed an accurate and high-performance approach SigmaCCS, which can integrate the advantages of the graph neural network (GNN) and the molecular graph including three-dimensional (3D) information for CCS prediction. The workflow of SigmaCCS is shown in Fig. 1. The SigmaCCS model was trained with the experimental CCS values from CCSbase^36^. The 3D conformers were generated by the experimental-torsion knowledge distance geometry method (ETKDG)^37^ and Merck molecular force field (MMFF94)^38^, which are good starting points for theoretical calculation and model-based prediction of CCS values. The molecular structures are encoded into molecular graphs, then fed into graph layers for higher-level feature extraction. The GNN is a suitable architecture for processing molecular graphs effectively. The performance of the model was evaluated by different metrics on the test set and the external test set. The importance of each atom attribute was investigated by a model-agnostic interpretation method, and the atomic degree, atomic symbol, is-in-ring, and 3D coordinates are attributes with relative importance larger than 10%. The learned representations are the pooled node attributes (**p**), and the visualization of the learned representations shows that they are chemically meaningful. The CCS values of compounds in PubChem were predicted, and an in-silico database with 282 million CCS values was generated.

## Results

### Hyperparameter optimization and training

**Fig. 2:**
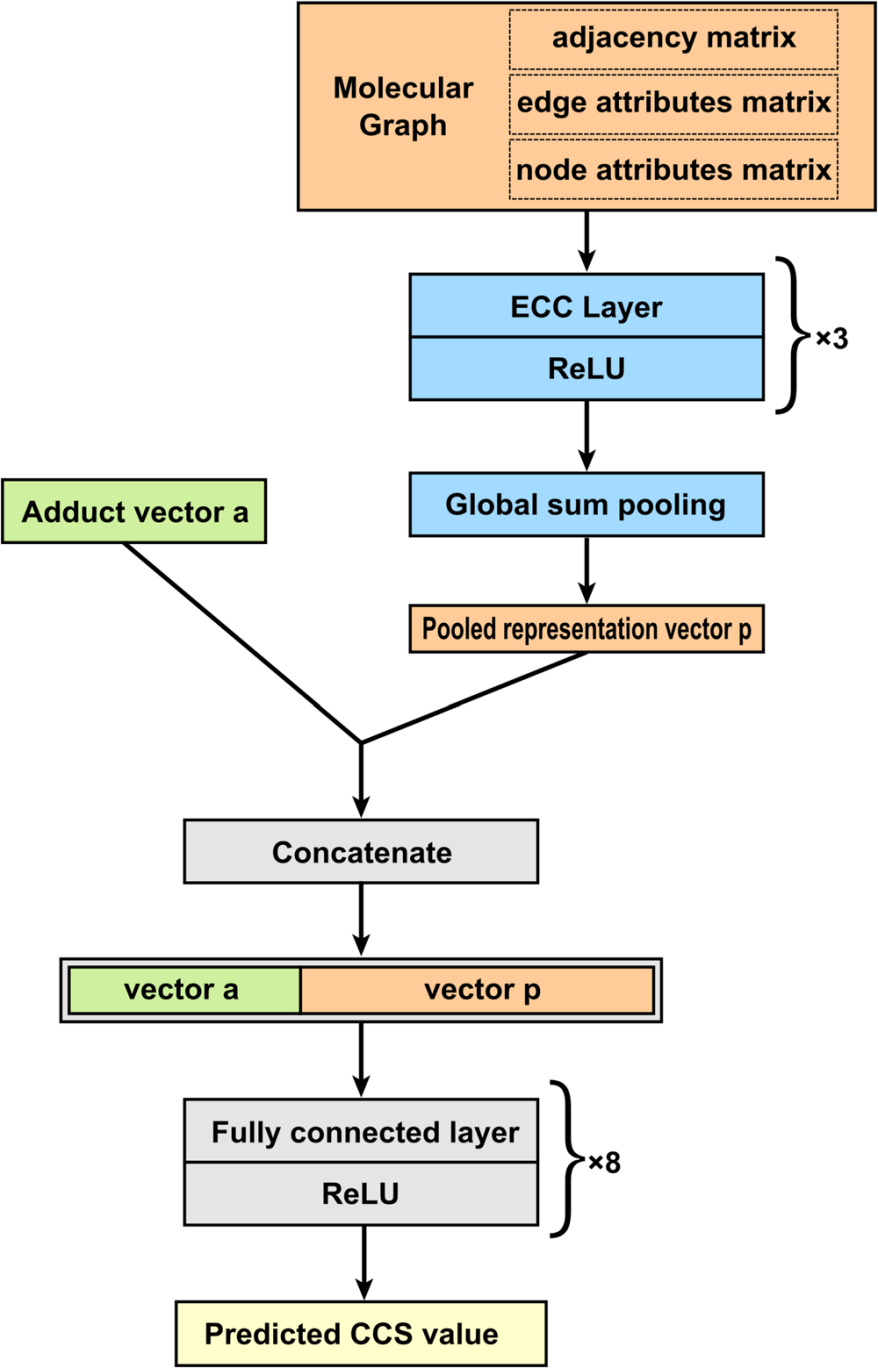
The neural network architecture of SigmaCCS. The ECC and global sum pooling are used to learn multilevel representations from molecular graphs. The CCS value can be predicted from the fused features by merging the learned representation with the one-hot encoding of an adduct ion.

The intuitive hyperparameters include the epoch, batch size, optimizer, learning rate, activation function, regularization method, the number of fully connected layers, and the number of units in fully connected layers. Their values optimized by the manual search are listed in Supplementary Table 1. The crucial hyperparameters are the type of graph convolutional layer and the number of graph layers. Their settings are listed in Supplementary Table 2 with six combinations in total. Five models were trained using the training subset for each hyperparameter combination, and the total number of trained models was 30. Their performance is evaluated on the validation subset listed in Supplementary Table 3. According to *R*^2^ and Median RE, the type of graph layer was chosen as edge-conditioned convolution (ECC)^39^, and the number of layers was set to 3. The neural network architecture of SigmaCCS is shown in Fig. 2. The loss curves of the training and validation subsets in the final training with the optimized hyperparameters are shown in Supplementary Fig. 1. The details of implementation and computing resources are presented in Supplementary Text 1.

### Performance evaluation

The training of deep learning models is often accompanied by some randomness. The effect of parameter initialization was explored by training ten different models with the training set. As listed in Supplementary Table 4, the standard deviation of *R*^2^ and Median RE on the test set are 0.0002 and 0.0795%, respectively. The results indicate that the effect of randomness in parameter initialization is small enough to negligible on the model performance. The performance of SigmaCCS on the test set based on molecular-level deduplication and adduct-level deduplication is listed in Supplementary Table 5. The result shows that the model performs excellently on the test set even after molecular-level deduplication.

**Fig. 3:**
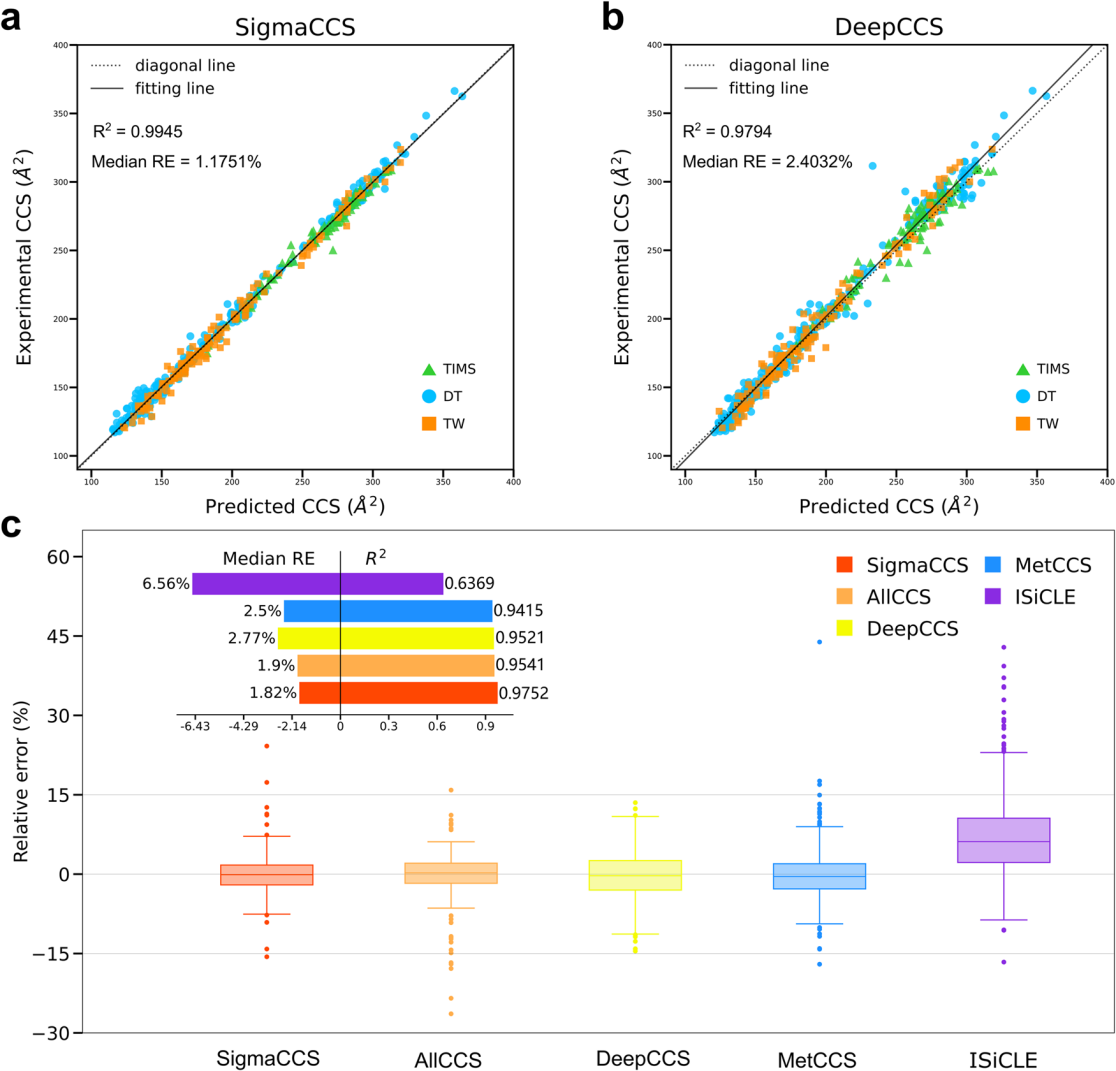
Performance evaluation of different methods. **a** SigmaCCS on the test set. **b** DeepCCS on the test set. **c** Performance comparison of SigmaCCS with AllCCS, MetCCS, DeepCCS, and ISiCLE on the external test set. 1.5 interquartile range was used as the error bars of the boxplot.

The SigmaCCS method was compared with DeepCCS proposed in 2019. The hyperparameters of SigmaCCS were set according to the previous section. The DeepCCS model was downloaded from its GitHub repository. Then, the SigmaCCS and DeepCCS models were evaluated on the test set. The results are listed in Supplementary Table 6. *R*^2^ and Median RE of SigmaCCS on the test set are 0.9945 and 1.175%, significantly better than the corresponding values of DeepCCS (0.9794 and 2.403%). The scatter plots of the experimental *vs*. predicted values of the test set are shown in Fig. 3a, b. All the points in the scatter plot of SigmaCCS are compact and close to the diagonal line. There is no systematic deviation between the fitting line and the diagonal line. All the points in the scatter plot of DeepCCS are more dispersed to the diagonal line when compared to SigmaCCS. Meanwhile, DeepCCS has a significant deviation of its predicted values from the experimental values at large CCS values. Furthermore, four molecules predicted by SigmaCCS resulted in the largest improvement compared to DeepCCS are listed in Supplementary Table 7. In the test set, the experimental CCS values are acquired by three different types of instruments, which are drift tube (DT), traveling wave (TW), and trapped ion (TIMS) IMS. The instrument types are plotted in different colors and shapes. As seen in Fig. 3a, SigmaCCS can reasonably predict the CCS values of these three different instruments with the same model.

The external test set was the dataset of AllCCS deduplicated by removing entries in the training set of SigmaCCS, and it was used to compare the performance of SigmaCCS with AllCCS, MetCCS, DeepCCS, and ISiCLE. As shown in Fig. 3c, SigmaCCS achieves the highest *R*^2^ and the lowest Median RE on the external test set. The scatter plots of the experimental *vs*. predicted values of SigmaCCS and DeepCCS on the external test set are shown in Supplementary Fig. 2. In short, SigmaCCS is an accurate and unbiased method to predict the CCS values.

Since there are 50 molecules of the external test set included in the training set of CCSbase, the external test set was further deduplicated by removing molecules in the training set of CCSbase. The number of CCS entries is 294 in the external test set. A disadvantage of Median RE is that it does not fully use all the data. Therefore, we introduce the root mean squared error (RMSE) as the metric to evaluate the performance of SigmaCCS and CCSbase. As shown in Supplementary Fig. 3a, b, *R*^2^, RMSE, and Median RE of SigmaCCS on the external test set are 0.9780, 6.7012, and 1.8211%, and the corresponding values of CCSbase are 0.9778, 6.7240, and 1.3608%. Furthermore, SigmaCCS is compared with CCSbase on the plant dataset^40^. As seen in Supplementary Fig. 3c, d, SigmaCCS (*R*^2^ = 0.9655, RMSE = 5.3812, and Median RE = 1.4232%) achieves better performances than CCSbase (*R*^2^ = 0.9643, RMSE = 5.4720, and Median RE = 2.3211%). The details of the performance evaluation of SigmaCCS and CCSbase on the external test set and the plant dataset are presented in Supplementary Text 2. CCSbase uses K-Means clustering for molecules and then trains a specific model for each cluster. As listed in Supplementary Table 8, molecules with large errors are further from the cluster centroids than those with small errors.

### Importance of atom attributes

**Fig. 4:**
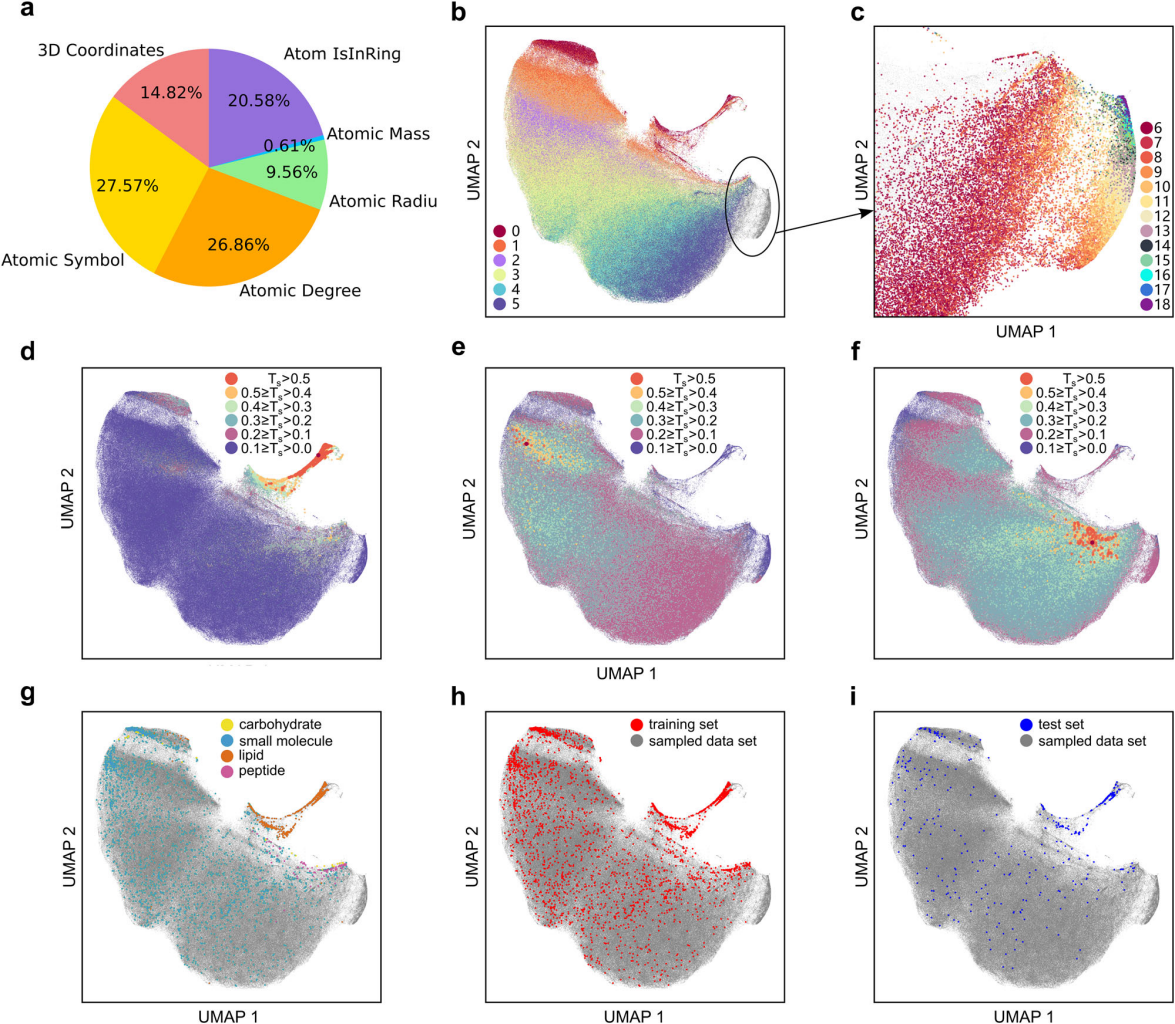
Visualization of the atom attribute importance and the learned representation. **a** The feature importance of atom attributes. **b** UMAP plot of learned representations colored by the ring number. **c** Zoomed UMAP plot on molecules with ring number greater than or equal to 6. **d**–**f** UMAP plots of molecules in the sampled data set colored by their Tanimoto similarities based on Morgan fingerprint to three randomly chosen molecules. **g** UMAP plot of all 3,996 molecules in the training and test sets colored by their molecular types. **h** UMAP plot of molecules in the training set and the sampled data set. **i** UMAP plot of molecules in the test set and the sampled data set.

The deep learning models are often regarded as black boxes lacking transparency and interpretability. Recently, some model-agnostic methods have been presented to interpret these black-box models. Among them, feature importance (FI) is a simple and popular method^41^. Its idea is simple: select one atomic attribute at a time, mask its values, and compare the performance difference between the masked model and the original model. If there is a significant drop in performance after masking the selected atom attribute, then that atom attribute is more significant. The FI of each attribute was calculated according to Supplementary Text 3. As shown in Fig. 4a, the FI of the atomic symbol (27.57%) is the highest because the structure of a molecule is mainly determined by the atoms and their connections. The FI of the atomic degree ranks second (26.86%). The possible reason is that the atomic degree describes the connections between atoms and is closely related to the topological structure of a molecule. The FI of the atomic radius is 9.56% because of the relationship between the atomic radius and atomic volume. The FI of the atomic mass is 0.61%, which is possible because their information is implicitly included in the atomic symbol. The FI of the "Atom IsInRing" ranks third because the ring structures may have a lower probability of collision and smaller CCS value than the linear molecules with the same atoms^14^. The FI of 3D coordinates (14.82%) ranks fourth. The CCS value is theoretically defined by averaging the projected areas of the 3D atomic spheres in different directions.

The 3D coordinates can directly reflect the projected areas. The CCS value of a molecule is closely related to its chemical structure and 3D conformation^12^. The 3D coordinates determine where there is a molecule, while the atom attributes (atomic symbol, degree, radius, mass, and atom IsInRing) determine the element types in 3D coordinates, bond angles, bond lengths, atomic radius, and the connections between atoms. These atom attributes also play an important role in the size and shape of the 3D conformers and thus impact the projected area of the 3D atomic sphere in different directions. The FI approach is widely used to interpret deep learning models. However, it only shows how the model makes use of the provided features without considering the interactions between the features. If a feature describes the same information as two other features implicitly do, the analysis only reveals the importance of the masked feature. The approach to interpretable machine learning needs further improvement. Meanwhile, it will be meaningful to assess the importance of combinations of features.

### Rotation of conformers

The details of the CCS prediction for the same molecule with different coordinates are presented in Supplementary Text 4. The 3D conformers of two randomly chosen molecules generated by ETKDG and MMFF94 are visualized in Supplementary Fig. 4. Histograms with fitted density curves for the predicted CCS values of the molecules with 1000 different 3D coordinates generated by ETKDG and MMFF94 are shown in Supplementary Fig. 5. Visualization of the 3D conformers of the molecule named 2,5-dihydroxybenzoic acid with completely random rotation is shown in Supplementary Figure 6. The performance of SigmaCCS on the test set and the external test set with different coordinates generated by ETKDG and MMFF94 is listed in Supplementary Table 9 and Supplementary Table 10, respectively. The performance on the test set with completely random rotation angles is listed in Supplementary Table 11. These results show that the model still performs well on CCS prediction even if the obtained coordinates of the same molecule have large rotations. Therefore, SigmaCCS is a reliable model, and there is no risk of overfitting with the 3D coordinates as the node attributes.

### Visualization of the learned representations

The pooled node attributes (**p**) are the learned representation of a molecule by the ECC layers and the global sum pooling layer. The dimensionality of **p** is 16. The uniform manifold approximation and projection (UMAP)^42^ can project the learned representations from 16 to 2 dimensions for visualizing and exploring.

Molecules in the training set and 1% randomly sampled compounds from PubChem (the sampled data set) were fed into SigmaCCS. Their pooled node attributes were extracted and visualized by UMAP. The number of rings in each molecule was calculated using RDKit. Each molecule was colored according to its ring numbers. From Fig. 4b, c, the molecules aggregate according to their ring numbers. There is a clear trend between the position of clusters and the ring numbers. These clustering results also further validated the rationality of the FI of the "Atom IsInRing" attribute in Fig. 4a.

We also randomly selected three target molecules from PubChem. Then, Tanimoto similarities (T_s_) were calculated based on Morgan fingerprints of molecules in the sampled data set and the target molecules. All the molecules in the sampled data set are colored by their similarities in Fig. 4d–f. The similarities decrease with increasing distances to the target molecules. Structurally similar molecules are clustered closer in UMAP plots based on the learned representations. It means that the learned representations correlate well with the molecular structures.

From the 5,597 entries in the training set and the test set, we extracted 3,996 molecules and their molecular classes. A UMAP model was fitted by the molecules in the sampled data set from PubChem. The molecules in the training and test sets were transformed into the learned space with the UMAP model. The molecules in the training and test sets are colored by their molecular classes in Fig. 4g. They are clustered according to their molecular classes (small molecules, lipids, peptides, and carbohydrates). Additionally, the molecules are also colored according to the training and test sets. As shown in Fig. 4h, i, the molecules in the training set and the test set are evenly distributed in the learned space of UMAP. The representations learned by ECC layers from molecular graphs have reasonable chemical significance. It lays a good foundation for the accurate prediction of CCS values.

### Relationship with the theoretical calculation

The ISiCLE is a pipeline proposed recently to calculate CCS values with the TM method, which can be used to verify the predicted values of SigmaCCS. The CCS values (825,702 entries of 281,318 molecules) calculated by ISiCLE (version 0.1.0) were downloaded from Pacific Northwest National Laboratory CCS database on July 29th, 2021.

**Fig. 5:**
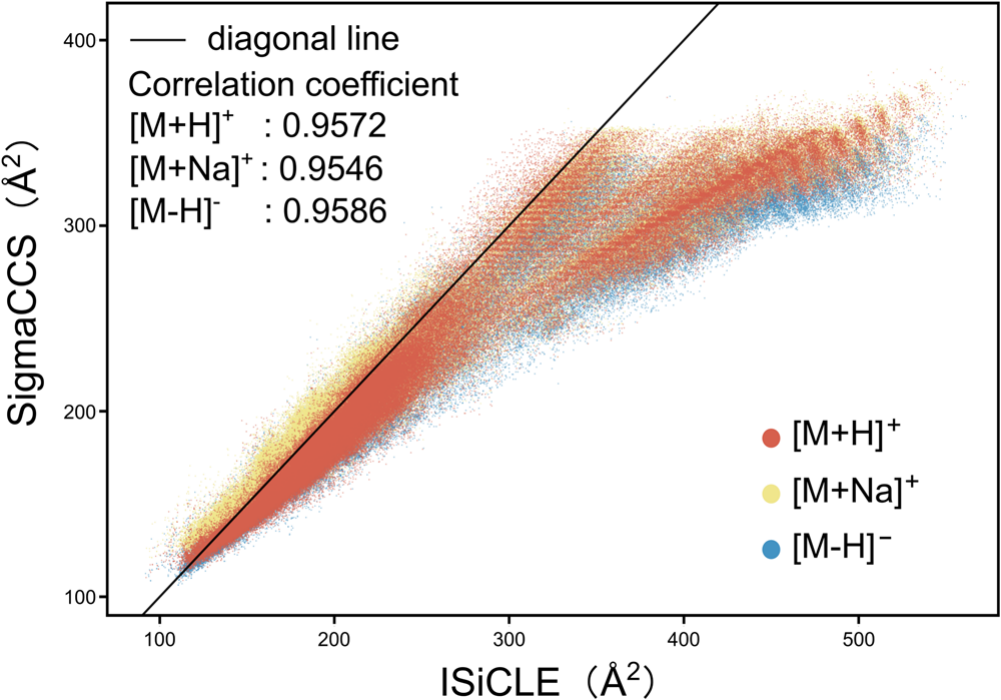
Comparison of the predicted CCS by SigmaCCS and the calculated CCS by ISiCLE. Visual representation of CCS values predicted by SigmaCCS and calculated by ISiCLE, colored by adduct types.

The calculated CCS values by ISiCLE and the predicted CCS values by SigmaCCS are shown in Fig. 5. There is a significant correlation between the calculated and predicted CCS values. The Pearson correlation coefficients are 0.9572, 0.9586, and 0.9546 for [M + H]^+^, [M+Na]^+^, and [M-H]^−^ adducts, respectively. The CCS values of ISiCLE are significantly larger than the values of SigmaCCS. This phenomenon is consistent with the results in Fig. 3c. There is an upper limit (around 380 Å^2^) of the CCS values predicted by SigmaCCS. By observing the CCS value distribution of the training set in Supplementary Data 1, the possible reason for the existence of this upper limit in the SigmaCCS model is that the number of molecules with CCS values greater than 380 Å^2^ is small in the training set (6 molecules in total, 5 peptides, and 1 carbohydrate). The performance of SigmaCCS on molecules with larger CCS values can be improved by pre-training with CCS values calculated by ISiCLE or (and) adding some molecules with larger experimental CCS values to the training set.

### CCS prediction of PubChem

The compounds in PubChem were chosen to build the in-silico CCS database with SigmaCCS. The upper limit of 380 Å^2^ was implicitly considered when performing the predictions on PubChem. The in-silico CCS database is established to assist in identifying the metabolites in organisms, which typically have molecular weights (MWs) under 1500 Dalton. Meanwhile, the CCS value of a molecule is closely related to its MW^43^. The experimental CCS *vs*. mass-to-charge ratio (*m/z*) for all adducts in the training and test sets of SigmaCCS is shown in Supplementary Fig. 7. It can be seen that experimental CCS and *m/z* are highly correlated, with Pearson correlation coefficients of 0.9813, 0.9748, and 0.9865 for [M + H]^+^, [M+Na]^+^, and [M-H]^−^ adducts, respectively. MW is an important physical property that is easy to obtain and does not require complex calculations. Therefore, molecules with MWs under 1500 Dalton are chosen from PubChem for CCS predictions. After filtering according to Supplementary Fig. 8 and conformer generation, there were 94,161,201 retained molecules. For each molecule, its CCS values of [M + H]^+^, [M+Na]^+^, and [M-H]^−^ adducts were predicted and filled in CSV files. These CSV files were uploaded to the Zenodo repository (10.5281/zenodo.5501673). According to the results in Fig. 4h, i, the filtered PubChem library is within the application domain of the SigmaCCS model, and the accuracy of the predicted CCS values is guaranteed for most entries. The details of in-silico CCS database generation are described in Supplementary Text 5.

### Multidimensional filtering assisted by SigmaCCS

The *m/z*, RTs, and CCS values of lipids were used together for multidimensional filtering with the assistance of the CCS values predicted by SigmaCCS. The mouse lung dataset was downloaded^44^, and 761 lipids in negative ion mode were chosen for multidimensional filtering. Meanwhile, LipidBlast was downloaded, and there were 256,696 retained entries in LipidBlast with negative ion mode. The list of candidates (MList) was retrieved from LipidBlast using the experimental *m/z* and the given threshold. Then, the candidates in the MList were filtered by matching the experimental and GNN-RT predicted RTs to obtain the RT-filtered list of candidates (RList). The candidates in the RList were further filtered by matching the experimental and predicted CCS values to obtain the CCS-filtered list of candidates (CList). Finally, the candidates in the CList were ranked according to their fused scores. More details of multidimensional filtering are presented in Supplementary Text 6. The results of the multidimensional lipid filtering are listed in Supplementary Table 12. Recall@1 increases from 15.2% to 24.6% and 28.9%, and recall@30 increases significantly from 47.6% to 78.4% and 91.2% when including *m/z*, RT, and CCS, gradually. It can be seen that this multidimensional matching procedure can improve the ranking of the correct molecule in the candidate list. The CCS values predicted by SigmaCCS are valuable for filtering false positives. In the case of isomeric compounds, the number of candidates at each filtering step and the ranking of the lipid (PubChem CID: 114944) are shown as an example in Supplementary Fig. 9.

## Discussion

The CCS values mainly depend on the 3D structures of molecules. By naturally encoding molecular structures into graphs, GNNs can process molecular graphs efficiently. In SigmaCCS, the ECC layers are mainly used for learning multilevel representations from molecular graphs. Then, the learned representations are fed to the fully connected layers to predict CCS values. The atom attributes (element types, degree, radius, atom IsInRing, mass, and 3D coordinates) are also the input of the neural network, and they are easy to obtain and do not require complex calculations. SigmaCCS can predict CCS values for molecules of any size end-to-end, using the basic attributes of atoms and bonds combined with a GNN. In general, the architecture of SigmaCCS is flexible and extendable. Although SigmaCCS cannot predict molecules of elements other than C, H, O, N, P, S, F, Cl, Br, I, Co, As, and Se, the molecule graph of the molecule consisting of other elements can be established by filling the corresponding columns of node attribute matrix with the attributes of the other elements. Meanwhile, the [M + H]^+^, [M+Na]^+^, and [M-H]^-^ are the three most common types of adducts in LC-MS analysis, and thus SigmaCCS focuses on the three adduct ion types. Further improvement can be made by encoding more adduct ion types in the one-hot vector. If enough molecules with different adduct ion types and different elements are available, the SigmaCCS model can be easily trained because of its flexibility, extendibility, and simplicity of inputs.

In comparison, DeepCCS feeds the one-hot encoding of SMILES string into CNN for representation learning. The input of most CNNs should be a tensor with fixed dimensions. Therefore, DeepCCS may fail on some SMILES strings beyond the training set. For example, the length of SMILES cannot be greater than 250 characters. In addition, DeepCCS can not perform a prediction when a SMILES string contains unknown chemical symbols. These problems limit the application scope of DeepCCS. There are 559 molecules in the test set. Only 514 molecules can be predicted by DeepCCS (Supplementary Table 6). AllCCS is a support vector regression-based method for CCS prediction using molecular descriptors. Its limitation is that molecular descriptors of a compound require complex calculations. CCSbase uses K-Means clustering for the untargeted classification of chemical structures and then performs CCS predictions using specific models trained on the corresponding cluster data. There exists a possibility of misclassification. If the molecule is assigned to an unsuitable cluster, it will make a relatively large deviation between the predicted CCS value and the experimental CCS value. ISiCLE is a theoretical calculation method. Although it is supported by solid theoretical principles, it requires complex computational steps, which makes a high demand on computational resources.

There are also some interesting findings in this study. First, the conformers of some molecules (33 out 94,161,896) in PubChem and (5 out 5645 entries) in CCSbase cannot be generated by the ETKDG method. There are 662 molecules whose conformers generated by ETKDG cannot be minimized by the MMFF94 method. Their structures are listed in Supplementary Data 2, which can be used to improve conformer generation and molecular force field methods. Second, the CCS values of SigmaCCS are significantly lower than the values of ISiCLE for molecules with large CCS values (>380 Å^2^). It can be attributed to the lack of molecules with high CCS values in the training set. Therefore, more molecules with large CCS values should be analyzed by IMS for training unbiased models in the entire domain. Third, the rationality of neural network architectures and the interpretability of the models significantly boost the confidence in the prediction results, which are recommended in similar studies.

## Methods

### Dataset curation

A high-quality dataset is a prerequisite for training deep neural networks with high accuracy. Therefore, the experimental database of CCSbase (V1.2) was downloaded from its official website. It includes 14,008 entries with measured CCS values by merging 22 datasets from 15 independent laboratories and 3 different types of instruments. The source, instrument type, size, and molecular category of each dataset are listed in Supplementary Table 13. Due to its diversity, a proper data curation procedure is required to improve the quality of the dataset for training, validating, and testing the SigmaCCS model. In this study, a data curation procedure consists of five steps: SMILES string verification, adduct type selection, the median of CCS values, unsuccessful conformation generation, and outlier removal. Their details can be found in Supplementary Text 7, and the outliers are listed in Supplementary Data 3.

After the above five filtering steps, the number of CCS entries was reduced from 14,008 to 5,597. The number of molecules is 3,996 in the curated dataset since one molecule may have multiple types of adducts. It shows vast chemical diversity with 17 superclasses, 104 classes, and 300 subclasses, according to the results of ClassyFire. The ClassyFire web service was not able to classify all the molecules. The classifiable molecules (3,365/3,996) are listed in Supplementary Data 4.

The curated dataset was divided into the training set (90%) and the test set (10%). The training set was further divided into the training subset (90%) and validation subset (10%) to tune the hyperparameters. Later, the training set and the optimized hyperparameters were used to train the SigmaCCS model. The test set was used to evaluate the performance of the trained model. The training subset, validation subset, and test set can be seen in Supplementary Data 1. There are 344 entries of 295 molecules in the external test set after deduplicating, which are listed in Supplementary Data 5. The plant dataset is from this article^40^, and there are 114 entries after deduplicating molecules in the training set of SigmaCCS, as listed in Supplementary Data 6. Its details are presented in Supplementary Text 2.

### Conformer generation

**Fig. 6:**
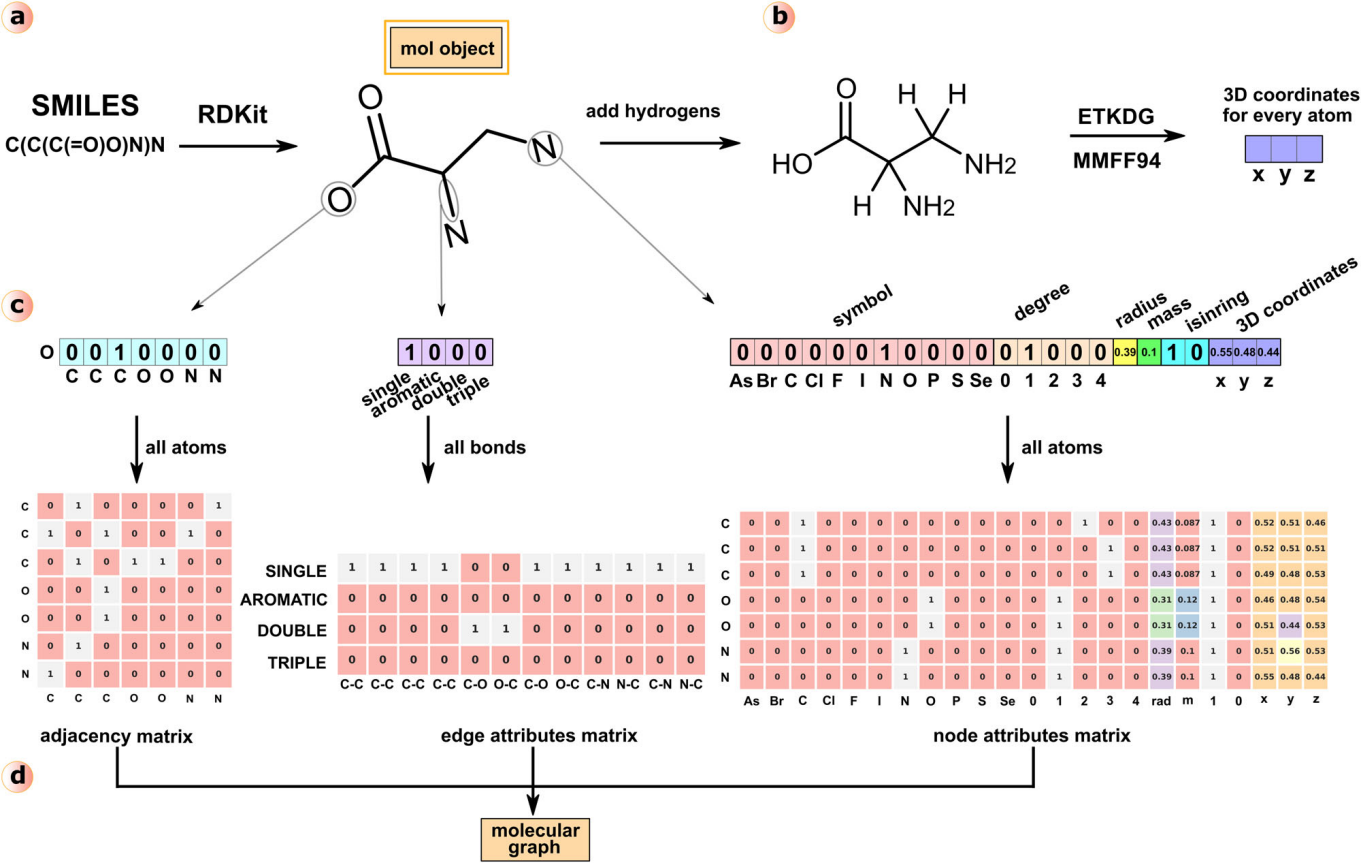
Schematic for constructing a molecular graph. **a** Conversion of the SMILES string into the RDKit Mol object. **b** Generation of 3D conformer with ETKDG and MMFF94. Coordinates of each atom were obtained from the 3D conformer. **c** Connection states, edge attributes, and node attributes of representative atoms and edges. Node attributes and edge attributes of all the atoms and bonds in the molecule were collected as node attributes matrix and edge attributes matrix. Connected states of all the atoms in the molecule were stored in the adjacency matrix. **d** Molecular graph consists of the node attributes matrix, edge attributes matrix, and adjacency matrix.

The CCS values are highly correlated with the 3D structures of molecules. The accuracy of model-based CCS prediction methods will be improved if the 3D structural information of molecules can be used as the input of deep neural networks. Efficient tools are needed to generate and optimize the 3D conformers of large-scale molecules from their SMILES strings. The ETKDG^37,45^ and MMFF94^38^ have been implemented in RDKit, which can generate conformers with distance geometry, correct the conformers with experimental-torsion knowledge, and optimize the conformers using molecular force fields. The ETKDG combined with MMFF94 is the best-performing freely available conformer generator^46^. It provides a quick way to generate 3D structures of molecules, which are suitable for the CCS prediction task. The workflow of conformer generation is shown in Fig. 6a, b.

### Molecular graph construction

A molecule can be naturally represented as a graph *G*(**v,** **e**). Here, **v**_i_ is the *i-*th atom, and **e**_*i,j*_ is the bond between the *i-*th and *j-*th atoms. The node attribute matrix **X** and the edge attribute matrix **E** are introduced to store the attributes of atoms and bonds, respectively. The adjacency matrix **A** is introduced to describe the connection of atoms in the molecule. In the curated dataset, molecular structural information was stored as SMILES strings. They were read as molecular objects by RDKit. Their 3D conformers were generated and optimized by ETKDG and MMFF94, respectively. Then, the atoms, bonds, and their attributes were obtained from these molecular objects. Attributes of each atom were filled into the corresponding row of node attributes matrix **X**. The detailed node attributes are shown in Fig. 6c and listed in Supplementary Table 14 and Supplementary Table 15. Attributes of each bond were filled into the corresponding row of edge attributes matrix **E**. The column size of **E** is four because there are four types of chemical bonds (single, double, triple, and aromatic) in the curated dataset. The adjacency matrix **A** was obtained by calling the GetAdjacencyMatrix function of the rdmolops module in RDKit with the molecular object. The constructed molecular graph is shown in Fig. 6c, d.

### Edge-conditioned convolution

The natural data structure for molecules is the molecular graph. The GNN^47,48^ is a type of neural network with the ability to operate on graph data structures, which has gained increasing popularity in the field of molecular property prediction. Recently, ECC was proposed by generalizing the convolution operator to graph and generate filter weights conditioned on edge attributes. Deep neural networks with ECC layers can handle datasets with varied graph sizes, apply graph convolutions to point clouds, and exploit the edge attributes. These advantages make it an ideal method for predicting molecular properties from their structures. Hence, the ECC layer is the core module to build a GNN-based method for CCS prediction. The ECC layer is formalized as follows:

1 $${{{{{{\bf{x}}}}}}}_{i}^{{{{{{\bf{o}}}}}}}={{{{{{\bf{x}}}}}}}_{i}{{{{{\bf{W}}}}}}+\mathop{\sum} \limits_{j\in {{{{{\rm{N}}}}}}(i)}{{{{{{\bf{x}}}}}}}_{j}{{\mbox{MLP}}}({{{{{{\bf{e}}}}}}}_{i,j})+{{{{{\bf{b}}}}}}$$

Here ${{{{{{\bf{x}}}}}}}_{i}$ and ${{{{{{\bf{x}}}}}}}_{i}^{{{{{{\bf{o}}}}}}}$ are the input and output of the ECC layer for the *i-*th atom. **W** is the trainable weight matrix. ${{{{{\rm{N}}}}}}(i)$ is the one-hop neighborhood(s) of the *i-*th atom. MLP is a multi-layer perceptron that outputs a bond-specific weight. ${{{{{{\bf{e}}}}}}}_{i,j}$ is attributes of the bond between *i-*th and *j-*th atoms. **b** is the trainable bias vector.

In this study, multiple ECC layers are used to learn multilevel representation from molecular structures. Given a molecular structure with *N* atoms, the output of the last ECC layer is a matrix ${{{{{{\bf{X}}}}}}}^{Last,{{{{{\bf{o}}}}}}}$ with *N* rows and *F* columns. The output of the last ECC layer should be used as the input of fully connected layers to predict the CCS value. However, the output size of the ECC layer varies with the number of atoms in the molecular graph. To solve this problem, the global sum pooling layer was introduced to pool a graph by adding node features across the node dimension. The global sum pooling for a graph *G* is computed by:

2 $${{{{{{\bf{p}}}}}}}_{j}=\mathop{\sum }\limits_{i=1}^{N}{{{{{{\bf{X}}}}}}}_{i,j}^{Last,{{{{{\bf{o}}}}}}}(j=1,2,{{{{\mathrm{..}}}}}.,F)$$

Here ${{{{{{\bf{X}}}}}}}^{Last,{{{{{\bf{o}}}}}}}$ is the output of the last ECC layer. **p** is the pooled representation of graph *G*, which is a vector of *F* elements.

### Adduct encoding

Electrospray ionization typically produces protonated [M + H]^+^ and deprotonated [M-H]^−^ ions in the positive and negative ion modes, respectively. Meanwhile, the adduct ion [M+Na]^+^ is also abundant for some molecules. The type of adduct significantly affects the CCS value of a molecule. Therefore, the type of adduct should be part of the input of the neural network. The SigmaCCS method focuses on the three most common types of adducts: [M + H]^+^, [M+Na]^+^, and [M-H]^−^. The one-hot encoding method is used to encode the type of adduct into the adduct vector (**a**) of size 3. The adducts [M + H]^+^, [M+Na]^+^, and [M-H]^−^ are encoded as [1, 0, 0], [0, 1, 0], and [0, 0, 1], respectively. Then, the pooled node attributes **p** and the adduct vector **a** are concatenated as the molecular vector **m** = [**p, a**], which is fed to fully connected layers.

### Fully connected layers

The fully connected layer has full connections to all activations of its previous layer, which is an efficient way to learn non-linear combinations of the learned representations and form the final output. Therefore, the last few layers of deep neural networks are usually fully connected layers. For this reason, the fully connected layers are used to process the molecular vector learned by the ECC layers and finally obtain CCS values in SigmaCCS. It can be described by the following equation:

3 $${{{{{{\bf{m}}}}}}}^{{{{{{\bf{o}}}}}}}=\sigma ({{{{{\bf{m}}}}}}{{{{{\bf{W}}}}}}+{{{{{\bf{b}}}}}})$$

Here, **m** is the molecular vector learned by ECC layers, and **m**^**o**^ is the output of fully connected layers. **W** and **b** are the learnable weights and bias, respectively. σ is the rectified linear unit (ReLU) activation function $\sigma (x)=\,\max (0,x)$.

### The architecture of SigmaCCS

The neural network input is the molecular graph consisting of the node attribute matrix, the edge attribute matrix, and the adjacency matrix constructed from the 3D conformer. The multilevel representations are learned from the molecular graphs with three ECC layers. The last ECC layer is followed by a global sum pooling layer, which gathers the learned representations from all the atoms in the molecule and outputs a pooled representation vector. The adduct type of the ion is encoded by the one-hot encoding method. The pooled representation and adduct vector are concatenated into the molecular vector. Then, eight fully connected layers are used to perform a nonlinear combination of the molecular vector to form the CCS value. For both the ECC layers and the fully connected layers, their activation functions are ReLU, and the L2 regularization is applied to the weights of these layers. The output layer is also a fully connected layer with a ReLU activation function but no regularization. The optimizer is Adam, which is a stochastic gradient descent method based on the adaptive estimation of first-order and second-order moments. The mean squared error is chosen as the loss function since it is appropriate for most regression problems.

To evaluate the performance of SigmaCCS on the CCS prediction, the metrics are presented in Supplementary Text 8.

## Supplementary information

Supplementary information

Description of Additional Supplementary Files

Supplementary Data 1

Supplementary Data 2

Supplementary Data 3

Supplementary Data 4

Supplementary Data 5

Supplementary Data 6

## Data Availability

The experimental database of CCSbase (V1.2) are available at its official website (https://ccsbase.net). The external test set is the dataset of AllCCS deduplicated by removing entries in the training set of SigmaCCS, which is provided in Supplementary Data 5. The plant dataset is from this article^40^, and there are 114 entries after deduplicating molecules in the training set of SigmaCCS, as listed in Supplementary Data 6. The molecules from PubChem to build the in-silico CCS database are available at the PubChem FTP site (https://ftp.ncbi.nlm.nih.gov/pubchem/Compound/CURRENT-Full/SDF). The predicted CCS values of compounds in PubChem can be downloaded from 10.5281/zenodo.5501673. The LipidBlast can be downloaded from MS-Dial software (http://prime.psc.riken.jp/compms/msdial/main.html). The mouse lung dataset can be downloaded from this article^44^. The predicted CCS values of compounds in LipidBlast are available on GitHub. The curated dataset for training, validating, and testing the SigmaCCS model is provided in Supplementary Data 1. Molecules in CCSbase and PubChem whose 3D conformers can not be generated or optimized by ETKDG and MMFF94 are available in Supplementary Data 2. Forty-three outliers detected in the curated dataset are listed in Supplementary Data 3. Chemical classification of the molecules in the curated dataset using the ClassyFire web service is provided in Supplementary Data 4.

## Code availability

The source code, models, document, manual, and tutorial are available on GitHub^49^ (https://github.com/zmzhang/SigmaCCS, https://github.com/yuxuanliao/SigmaCCS, and https://github.com/YoujiaZhang/SigmaCCS).
