# Supplementary material for: Highly accurate and large-scale collision cross sections prediction with graph neural networks
Source: Commun Chem. 2023 Jul 4;6:139. doi: 10.1038/s42004-023-00939-w (PMC10319785; doi:10.1038/s42004-023-00939-w)
Supplement: Supplementary file 1 — Supplementary information [file 42004_2023_939_MOESM1_ESM.pdf]

Supplementary information

Highly accurate and large-scale collision cross sections prediction with graph neural networks

Renfeng Guo[1,†], Youjia Zhang[2,†], Yuxuan Liao[1,†], Qiong Yang[1], Ting Xie[1], Xiaqiong Fan[1], Zhonglong Lin[3], Yi Chen[3,*], Hongmei Lu[1,*], Zhimin Zhang[1,*]

[1] College of Chemistry and Chemical Engineering, Central South University, Changsha 410083, China.

[2] School of Computer Science and Technology, Huazhong University of Science and Technology, Wuhan 430074, China.

[3] Yunnan Academy of Tobacco Agricultural Sciences, Kunming, Yunnan 650021, China.

[†] These authors contributed equally.
[*] These authors jointly supervised this work.

[*] Correspondence: cytobacco007@sina.com, hongmeilu@csu.edu.cn, and zmzhang@csu.edu.cn

List of Supplementary Texts

Supplementary Text 1. Details of implementation and computing resources

Supplementary Text 2. Details of performance evaluation of SigmaCCS and CCSbase on the external test set and the plant dataset

Supplementary Text 3. The feature importance method

Supplementary Text 4. CCS prediction for the same molecule with different coordinates

Supplementary Text 5. in-silico CCS database generation

Supplementary Text 6. Multidimensional filtering assisted by SigmaCCS

Supplementary Text 7. Details of dataset curation

Supplementary Text 8. Details of evaluation metrics

List of Supplementary Figures

Supplementary Figure 1. The loss curves of the training and validation subsets in the final training with the optimized hyperparameters

Supplementary Figure 2. Performance evaluation of SigmaCCS and DeepCCS on the external test set

Supplementary Figure 3. Performance evaluation of SigmaCCS and CCSbase

Supplementary Figure 4. Visualization of the 3D conformers of two randomly chosen molecules generated by ETKDG and MMFF94

Supplementary Figure 5. Histograms with fitted density curves for the predicted CCS values of the molecules with 1000 different 3D coordinates generated by ETKDG and MMFF94

Supplementary Figure 6. Visualization of the 3D conformers of the molecule named 2,5-dihydroxybenzoic acid with completely random rotation

Supplementary Figure 7. Visual representation of experimental CCS *vs*. *m/z* for all adducts in the training and test sets of SigmaCCS

Supplementary Figure 8. Extraction and selection of proper molecules from PubChem to build the *in-silico* CCS database

Supplementary Figure 9. Multidimensional filtering assisted by SigmaCCS

List of Supplementary Tables

Supplementary Table 1. Settings for some intuitive hyperparameters

Supplementary Table 2. Different combinations of two crucial hyperparameters

Supplementary Table 3. The performance of the models with different hyperparameters on the validation subset

Supplementary Table 4. Evaluation of the randomness in parameter initialization on the performance of the models on the test set

Supplementary Table 5. Performance of SigmaCCS on the test set and the test set after molecular-level deduplication

Supplementary Table 6. Comparison of SigmaCCS and DeepCCS on the test set

Supplementary Table 7. Four molecules predicted by SigmaCCS resulted in the largest improvement compared to DeepCCS

Supplementary Table 8. The 3 molecules with the largest relative error and the 3 molecules with the smallest relative error and their distances to the cluster centroids

Supplementary Table 9. Performance of SigmaCCS on the test set with different coordinates generated by ETKDG and MMFF94

Supplementary Table 10. Performance of SigmaCCS on the external test set with different coordinates generated by ETKDG and MMFF94

Supplementary Table 11. Performance of SigmaCCS on the test set with completely random rotation angles

Supplementary Table 12. Results of the multidimensional lipid filtering

Supplementary Table 13. Source of experimental data sets

Supplementary Table 14. Attributes of nodes (atoms), edges (chemical bonds), and ion types

Supplementary Table 15. The atomic mass and radius used to construct molecular graphs

Supplementary Text 1. Details of implementation and computing resources

All the methods were implemented in Python (v3.7.7). The dataset curation was done using Pandas (v1.2.5) and RDKit (v2020.09.5.0). Conformers were generated by ETKDG and MMFF94 in RDKit. The implementation of GNN was based on Tensorflow (v2.4.0-GPU) and spektral (v1.0.5). All the computations were submitted to the Inspur TS10000 HPC cluster of Central South University. For the training of the SigmaCCS model, the allocated node was a GPU node with 2 Intel(R) Xeon(R) Gold 6248R processors, 2 Nvidia Tesla V100s, and 384G DDR4 memory. For the large-scale prediction of CCS values, a total of 25 CPU nodes were allocated. Each CPU node includes 2 Intel(R) Xeon(R) Gold 6248 processors and 192G DDR4 memory.

Supplementary Text 2. Details of performance evaluation of SigmaCCS and CCSbase on the external test set and the plant dataset

The external test set and the plant dataset were used to compare the performance of SigmaCCS with CCSbase.

(**a**). **Performance evaluation on the external test set.** We investigate whether the training set of CCSbase contains the molecules in the external test set. Since the prediction model (V1.2) of CCSbase is used to make a comparison with SigmaCCS, the experimental database (V1.3) for training the prediction model (V1.2) was downloaded from its official website. The training set of CCSbase is obtained by data splitting using the random seed described in the article[1]. There are 50 molecules of the external test set included in the training set of CCSbase. Then, the external test set was further deduplicated by removing molecules in the training set of CCSbase. The number of CCS entries is 294 in the external test set. As shown in Supplementary Figure 3a and 3b, R^2 and Median RE of SigmaCCS on the external test set are 0.9780 and 1.8211%, and R^2 and Median RE of CCSbase on the external test set are 0.9778 and 1.3608%. A disadvantage of Median RE is that it does not fully use all the data. Therefore, the root mean squared error (RMSE) is used as the metric to evaluate the performance of SigmaCCS and CCSbase. The RMSE of SigmaCCS on the external test set is 6.7019, which is better than the corresponding value of CCSbase (6.7240). In addition, the number of molecules with relative errors larger than 8% based on the predicted CCS values of SigmaCCS and CCSbase is 6 and 9, respectively. CCSbase first uses K-Means clustering for the untargeted classification of chemical structures and then performs CCS predictions using specific models trained on the corresponding cluster data. We selected three molecules with the largest relative error and three with the smallest relative error of CCSbase, converted the molecules into numerical representation based on structural features (MQNs, MS adduct, and *m/z*), and then calculated the distances to the centroid of each cluster. As listed in Supplementary Table 8, molecules with large errors are further from the cluster centroids than those with small errors. There exists a possibility of misclassification by K-Means. If the molecule is assigned to an unsuitable cluster, it will make a relatively large deviation between the predicted CCS value and the experimental CCS value.

(b). Performance evaluation on the plant dataset. The plant dataset of 146 natural plant products[2] was used to compare SigmaCCS and CCSbase fairly. After removing unpredictable adducts and deduplicating molecules in the training set of SigmaCCS, the number of CCS entries is 114 in the plant dataset. There is a molecule (Compound CID: 98775) whose relative error is 29.75% based on the predicted value of CCSbase. Therefore, the molecule is regarded as an outlier and has been removed from the plant dataset. The size of the plant dataset was reduced to 113. The scatter plots of the experimental *vs*. predicted values of SigmaCCS and CCSbase on the plant dataset are shown in Supplementary Figure 3c and 3d, respectively. It can be seen that SigmaCCS (R^2 = 0.9655, RMSE = 5.3812, and Median RE = 1.4232%) achieves better performances than CCSbase (R^2 = 0.9643, RMSE = 5.4720, and Median RE = 2.3211%).

Supplementary Text 3. The feature importance method

Here, the feature importance method was used to investigate the importance of each atom attribute on the CCS prediction performance of SigmaCCS. First, the original model was built, and its mean absolute percentage error (MAPE) on the test set was calculated as $MAPE^{orig}$. Second, the mask was used to cover the j-th feature bits on the atoms to make them invalid. The modified molecular graphs were used to predict the CCS values and calculate the $MAPE^{mask}$. Then, the feature importance (FI) of the j-th atom attribute was calculated by:

$$\mathrm{MAPE} = \frac{100}{n}\sum_{i=1}^{n}\left|\frac{y_i - \hat{y}_i}{y_i}\right| \tag{S1}$$

$$\mathrm{FI}_j = \frac{MAPE^{orig} - MAPE_j^{mask}}{\sum_{j=1}^{F}(MAPE^{orig} - MAPE_j^{mask})} \tag{S2}$$

Here, y_i is the experimental value. *n* is the number of molecules in the test set, and *F* is the number of atom attributes. Finally, the FI values of all the atom attributes were calculated using equation **(S2)** by averaging the prediction results of 10 models.

Supplementary Text 4. CCS prediction for the same molecule with different coordinates

The CCS value of a molecule is closely related to its chemical structure and three-dimensional conformation[3]. The 3D coordinates of a molecule contain structural information. In this study, SigmaCCS includes 3D coordinates into atom feature vectors as additional information reflecting the molecular structure. To verify that the lacking invariance of 3D coordinates does not lead to a risk of overfitting, we have performed the following experiments.

There is an optional parameter (randomSeed) when using ETKDG to obtain the 3D coordinates of a molecule. By setting different seeds, different coordinates can be obtained for a molecule on multiple runs. Two molecules were randomly selected from the test set as examples, and 2, 10, 100, and 1000 conformers were generated for each molecule using different seeds of ETKDG and MMFF94. Their 3D conformers are visualized in Supplementary Figure 4. ETKDG uses distance geometry to obtain the 3D coordinates of each atom in a molecule. Since the distance geometry places the center of mass of the molecule at the origin of the coordinates[4], there are no shifts in 3D conformers of the same molecule generated by ETKDG. It can be seen from the aggregation of 1000 conformers of the same molecule into a sphere in Supplementary Figure 4. Since the data sets of SigmaCCS are processed by ETKDG, there is no shift problem in this study.

As shown in Supplementary Figure 4a and 4b, the 3D coordinates of the conformers obtained using ETKDG and MMFF94 have large rotations instead of small random disturbances for the same molecule. The 1000 conformers of the two molecules were fed into SigmaCCS to predict their CCS values. The distributions of the predicted CCS values of the two molecules are shown in Supplementary Figure 5a and 5b, respectively. The mean value and standard deviation of the predicted CCS values of 2,5-dihydroxybenzoid acid with 1000 different 3D coordinates are 122.63 Å^2 and 0.246 Å^2, respectively. The mean value and standard deviation of the predicted CCS values of Praziquantel with 1000 different 3D coordinates are 176.41 Å^2 and 0.228 Å^2, respectively. It can be seen that the predicted CCS values are stable using conformers with large rotations as inputs.

We performed 30 batches of CCS predictions for the test set using different seeds of ETKDG. The performance of SigmaCCS on the test set with different coordinates is listed in Supplementary Table 9. The standard deviation of R^2 and Median RE on the test set are 0.00008 and 0.0269%, respectively.

In addition, we performed the same experiment on the external test set. The performance of the model is evaluated on the external test set with different coordinates obtained by ETKDG and MMFF94 listed in Supplementary Table 10. The standard deviation of R^2 and Median RE on the external test set are 0.00028 and 0.0445%, respectively. These results show that the model still performs well on CCS prediction even if the obtained coordinates of the same molecule have large rotations. Therefore, SigmaCCS is a reliable model, and there is no risk of overfitting with the 3D coordinates as the node attributes.

Since the 3D coordinates of all molecules in the training and test sets are generated using ETKDG and MMFF94, the conformers of the same molecule gradually cluster into the sphere with the increasing number of conformers, as visualized in Supplementary Figure 4. Therefore, we rotated each molecule in the test set to a completely random position to evaluate the performance on the test set with different rotation angles. As shown in Supplementary Figure 6, the 3D conformer of the molecule (PubChem CID: 3469) is rotated around the x, y, and z-axes by random angles, respectively. The 3D conformer of each molecule in the test set was generated using ETKDG and MMFF94. Then, it was rotated to a completely random position around the x, y, and z-axes using its random rotation matrix, respectively. The rotation angle of each molecule in the test set is completely random. As listed in Supplementary Table 11, the standard deviation of R^2 and Median RE on the test set are 0.00006 and 0.0213%, respectively. There is no performance drop on the test set with completely random rotation angles. The result shows that any rotational position work as good as the initial position.

Supplementary Text 5. in-silico CCS database generation

A structural database with CCS values predicted by SigmaCCS should be established to assist in identifying the compound. It mainly consists of four steps, including molecular selection, CCS value prediction, database schema definition, and creation.

Molecules in the structural database should be selected to meet the requirements of both models and application fields. In this study, we focus on predicting the CCS values of compounds in organisms by SigmaCCS. Therefore, several rules are defined to filter out molecules that unlikely present in organisms and could not be predicted by SigmaCCS. First, the molecule must contain both carbon and hydrogen atoms, which can guarantee that the molecules chosen are highly possible to be organic compounds. Second, the elements of each selected molecule must be a subset of all the elements (C, H, O, N, P, S, F, Cl, Br, I, Co, As, and Se) in the training set. Third, the selected molecules should not contain ionic bonds, and this is because the training set of SigmaCCS does not include ionic bonds. Furthermore, the other special requirements for the selected molecules can also be set as filter rules according to the application fields or the models.

Molecular structure databases are usually large and often have millions of molecules even after filtering by the above rules. Each molecule should go through the steps of 3D conformer generation, molecular graph construction, and SigmaCCS prediction to obtain its CCS values. Although each step is relatively fast and does not take much time for a single molecule (0.4s for conformer, 0.0008s for graph, and 0.013s for prediction on CPU). When applied to large-scale molecules, theoretically, the prediction procedure would take 5 days for one million molecules and 500 days for 100 million molecules. By integrating these three steps into a function and parallelizing the function with the multiprocessing package in Python, multi-core computing can be leveraged to accelerate the prediction process. For one million molecules, about 30 times acceleration can be achieved using a single CPU node (48 cores), and the CCS value prediction for all molecules can be finished within 5 hours. For 100 million molecules, about 500 times acceleration can be achieved by splitting and assigning the computational task to 25 CPU nodes (48×25=1200 cores) using the Slurm workload manager, and the CCS prediction for all molecules can be finished within two days. The number of nodes can be adjusted according to the number of selected molecules and the acceptable time of the prediction task.

The creation of a database requires a well-defined schema. After thorough consideration of the properties of molecules, the following fields are chosen to store in the database: molecular identifier in the original database, InChi identifier, InChIKey, SMILES string, formula, molecular weight, predicted CCS values of $[M+H]^+$, $[M+Na]^+$ and $[M-H]^-$. Then, the database and table can be created according to the defined schema in any database engine (SQLite, MySQL, etc.). For all the selected molecules, their relevant information and predicted CCS values are stored in standard CSV format during the prediction procedure, which can be imported into the database in batches. It is worth mentioning that the combined index can be created on the columns of molecular weight and predicted CCS values to accelerate the retrieval procedure of candidates.

The application of CCS values in compound identification requires the prediction of CCS values for a large number of molecules to improve compound coverages. PubChem is the largest collection of freely accessible chemical information[5, 6, 7]. In the compound database of PubChem, there are 110 million entries of compounds with names, identifiers, structures, physicochemical properties, spectral information, etc. Therefore, the compounds in the compound database of PubChem were chosen to build the *in-silico* CCS database with SigmaCCS. The PubChem database was downloaded from its FTP site in structure data format (SDF) on May 6th, 2021. There were 314 SDF files containing 110 million entries. The ID, InChi, InChikey, SMILES, formula, and molecular weight were parsed from the SDF files and saved as CSV files. After filtering with criteria of hydrogen & carbon, elements, bond, mass & number of atoms, isotopes & duplication, there were 94,161,896 retained entries. The procedure of extracting and selecting proper molecules from PubChem to build the *in-silico* CCS database is shown in Supplementary Figure 7.

High-performance computing (HPC) is crucial to scientific research with big data in chemistry. The time can be reduced from years to days when migrating the computing tasks from a personal computer to an HPC cluster. Twenty-five CPU nodes with 1200 cores were allocated in the HPC cluster of

Central South University to accelerate 3D conformer generation, molecular graph construction, and CCS prediction. The computational tasks were evenly distributed among these 25 nodes. All the computing tasks were completed within two days. The ETKDG and MMFF94 were used to generate 3D conformers for all 94,161,896 molecules. The 3D conformer generation failed for 695 molecules. Among them, 33 molecules attributed to ETKDG and 662 molecules to MMFF94. Their SMILES, InChi, InChikey, PubChem ID, source, and reason are listed in Supplementary Data 2. The number of remaining molecules in the *in-silico* CCS database is 94,161,201. For each molecule, its predicted CCS values of $[M+H]^+$, $[M+Na]^+$, and $[M-H]^-$ adducts were filled in the CSV files. Then, these CSV files were uploaded to the Zenodo open-access repository (https://doi.org/10.5281/zenodo.5501673).

Supplementary Text 6. Multidimensional filtering assisted by SigmaCCS

The CCS values derived from ion mobility spectrometry (IMS) can be used to improve the accuracy of compound identification. The starting point for multidimensional filtering is the acquired data (*m/z*, RT, and CCS) of the compound to be identified and an *in-silico* CCS database. Therefore, we downloaded the mouse lung dataset from this article[8], and there are 2,070 lipids with the *m/z*, RT, and CCS information in the mouse lung dataset. After the removal of unpredictable adducts and empty SMILES strings, 761 lipids are in negative ion mode, and 262 lipids are in positive ion mode. Since more lipids need to be identified in negative ion mode than in positive ion mode, the negative ion mode is chosen for multidimensional filtering. Meanwhile, LipidBlast was downloaded from its official website. The dataset with negative ion mode has a total of 356,477 molecules of 94 classes. After removing unpredictable adducts, there are 256,696 retained entries. The CCS values of the compounds were predicted by SigmaCCS to build the *in-silico* CCS database. Predicting RT from the molecular structure is a difficult task because it is susceptible to experimental conditions. With the GNN-RT method[9] and the transfer learning technique[10], the pre-trained GNN-RT model can be transferred to the target chromatographic system using only about 100 molecules with experimental RTs. The CCS filter is used as the final filtering step to show the performance of CCS values predicted by SigmaCCS for multidimensional filtering. The reason for not using information from the MS/MS dimension is that there is no suitable prediction method for tandem mass spectra of lipids. The detailed process of multidimensional filtering is as follows:

(**a**). **Filtering with m/z.** For each molecule in the LipidBlast dataset, its *m/z* was stored as precursor *m/z*. The list of the candidate molecules (*MList*) can be retrieved from the LipidBlast dataset with the experimental *m/z* and the *m/z* threshold (t_m). When t_m was set to 30 ppm, the false negative rate was 0.31%. The scoring function for *m/z* is defined as follows:

$$S_m = \begin{cases} 1, & M \le M_{\min} \\ 1 - \dfrac{M - M_{\min}}{M_{\max} - M_{\min}}, & M_{\min} < M < M_{\max} \\ 0, & M \ge M_{\max} \end{cases} \tag{S3}$$

Here M is the relative error between the *m/z* of adduct ions in the candidate list and the *m/z* of adduct ions calculated by RDKit. M_{max} and M_{min} are the maximum and minimum mass errors, respectively. The default values for M_{max} and M_{min} are 20 ppm and 50 ppm, respectively.

(**b**). **Filtering with m/z and retention time.** The GNN-RT model was pre-trained by the SMRT dataset[11] (80,038 small molecules with experimental RTs) and transferred to the MS-DIAL 4 dataset containing 4,303 molecules of 108 lipid subclasses. The transferred model can achieve good performance in retention time prediction for the lipid chromatographic system. The RT-filtered list of candidates (*RList*) was obtained by eliminating the molecules in the *MList* using the experimental RT and the RT filtering threshold (t_r). When t_r was set as the relative error of 20%, the false negative rate was 1.1858%. The scoring function for RT is defined as follows:

$$S_r = \begin{cases} 1, & R \le R_{\min} \\ 1 - \dfrac{R - R_{\min}}{R_{\max} - R_{\min}}, & R_{\min} < R < R_{\max} \\ 0, & R \ge R_{\max} \end{cases} \tag{S4}$$

Here R is the relative error between the predicted and measured RTs. R_{max} and R_{min} are the maximum and minimum relative errors in the GNN-RT test set.

(**c**). **Filtering with m/z, retention time and CCS.** The CCS values of all the candidates in the *RList* were predicted by SigmaCCS. Similarly, if the relative error of a candidate is larger than a given threshold, it should be eliminated from the candidate list as a false positive candidate. The CCS-filtered list of candidates (*CList*) was obtained by eliminating the molecules in the *RList* by comparing the relative error between the experimental and predicted CCS values with the CCS filtering threshold (t_c). When t_c was set as the relative error of 5%, the false negative rate was 0.9%. The scoring function for CCS values is defined as follows:

$$S_c = \begin{cases} 1, & C \le C_{\min} \\ 1 - \dfrac{C - C_{\min}}{C_{\max} - C_{\min}}, & C_{\min} < C < C_{\max} \\ 0, & C \ge C_{\max} \end{cases} \tag{S5}$$

Here C is the relative error between the predicted and measured CCS values. C_{max} and C_{min} are the maximum and minimum relative errors in the test set of SigmaCCS, respectively. The relative error of *m/z*, RT, and CCS can be calculated as follows:

$$RE = \frac{\left| X_{pred} - X_{\exp} \right|}{X_{\exp}} \tag{S6}$$

X_{pred} is the predicted value of the candidate molecule, and X_{exp} is the measured value of the component to be identified. To quantitatively analyze the matching degree between the predicted and experimental values, the fused score of a candidate molecule is calculated as follows:

$$S_f = W_m \times S_m + W_r \times S_r + W_c \times S_c \tag{S7}$$

Here W_m, W_r, and W_c are the weights for *m/z*, RT, and CCS scores. The score function weights for *m/z*, RT, and CCS were set as 0.6, 0.2, and 0.2, respectively. Finally, the candidate molecules are ranked according to their fused scores for multidimensional filtering.

The sorted *CList* was obtained by ranking the candidates in the *CList* according to their fused scores in descending order. The ranking of each lipid was analyzed, and recall@1, recall@10, recall@20, recall@30, and recall@40 were 28.9%, 63.5%, 79.6%, 91.2%, and 94.7%, respectively. Results of the lipids filtering with the *m/z*, *m/z* + RT, and *m/z* + RT + CCS are listed in Supplementary Table 8. Recall@1 increases from 15.2% to 24.6% and 28.9%, and recall@30 increases significantly from 47.6% to 78.4% and 91.2% when including *m/z*, RT, and CCS, gradually. It can be seen that the rankings of the correct molecules increase after each filtering step. The CCS values predicted by SigmaCCS are valuable for filtering false positives. Furthermore, in the case of isomeric compounds, the lipid (PubChem CID: 114944) was identified as an example. The number of candidates at each step and the ranking of the lipid are shown in Supplementary Figure 8. Results show that the ranking of the compound is 49th in the *MList* of 88 candidates, the ranking of the compound is 5th in the *RList* of 55 candidates, and the ranking of the compound is 1^{sh} in the *CList* of 55 candidates. In short, the predicted CCS values by SigmaCCS, along with accurate *m/z* and RTs of compounds, were fused together to improve the accuracy of compound identification. When searching large structural libraries for compound identification, the application of multidimensional information can efficiently remove false positive compounds and significantly improve the accuracy of identification.

Supplementary Text 7. Details of dataset curation

SMILES string verification: The entries without SIMILES strings were removed from the dataset. The "." in SMILES strings indicates that two molecular parts are dissociated and not bonded together. These entries were also deleted from the dataset. After this step, the dataset size was reduced to 11,813.

Adduct type selection: The $[M+H]^+$, $[M+Na]^+$, and $[M-H]^-$ are the three most common types of adducts in LC-MS analysis. Consequently, the entries of $[M+H]^+$, $[M+Na]^+$, and $[M-H]^-$ adduct types were retained in the dataset, and all the entries of other adduct types were removed from the dataset. After this step, the dataset size was reduced to 8,829.

Median of CCS values: The CCSbase was created by merging multiple datasets. There exists the same adduct with multiple different CCS values. To solve this problem, the Median of the multiple CCS values was taken as the CCS value for this adduct. After this step, the dataset size was reduced to 5,645.

Unsuccessful conformation generation: The 3D conformers are good starting points for theoretical calculation and model-based prediction of CCS values. Here, the conformer generator and molecular force field in RDKit were used to construct the 3D conformer of each molecule from its SMILES string and optimize the conformation. There are a tiny number of molecules (5 in 5,645 entries) whose conformations cannot be generated by RDKit even after trying three times. They are listed in Supplementary Data 2, and the values of the source column are marked as CCSbase. Those five molecules were eliminated from the dataset. The dataset size was reduced to 5,640.

Outlier removal: In DeepCCS, CCS prediction tools have been shown to be potentially useful for database validation. Suspect measurements can be detected by comparing predicted and experimental CCS values. Most models of CCS prediction have a median relative error (Median RE) of around 2%. According to 5 × Median RE, if the relative error of a molecule is larger than 10%, it is classified as an outlier. Since the training set of SigmaCCS was obtained from CCSbase, we compared the CCS values predicted by CCSbase with measured CCS values to remove outliers. There were 43 molecules whose relative error larger than 10% based on the predicted values of CCSbase. It was also found that the CCS values of some molecules did not match with the SMILES strings by comparing the SMILES string provided by CCSbase and the formula provided in the corresponding source. It further illustrates the rationality of outlier removal. All 43 molecules are listed in Supplementary Data 3. These 43 molecules were removed from the dataset, and the dataset size was reduced to 5,597.

Supplementary Text 8. Details of evaluation metrics

To evaluate the performance of SigmaCCS on the CCS prediction, the metrics used in this study are the coefficient of determination (R^2) and the median relative error (Median RE). The R^2 and Median RE are defined as follows:

$$R^2 = 1 - \frac{\sum_{i=1}^{n}\left(y_i - \hat{y}_i\right)^2}{\sum_{i=1}^{n}\left(y_i - \bar{y}\right)^2} \tag{S8}$$

$$Median\ \ RE = median\left(\frac{\left|\hat{y}_i - y_i\right|}{y_i}\right) \tag{S9}$$

Here y_i and $\hat{y}_i$ are the experimental and predicted CCS values of the *i*-th molecule. $\bar{y}$ is the mean of the experimental CCS values in the test set or the external test set. *n* is the number of molecules in the dataset.

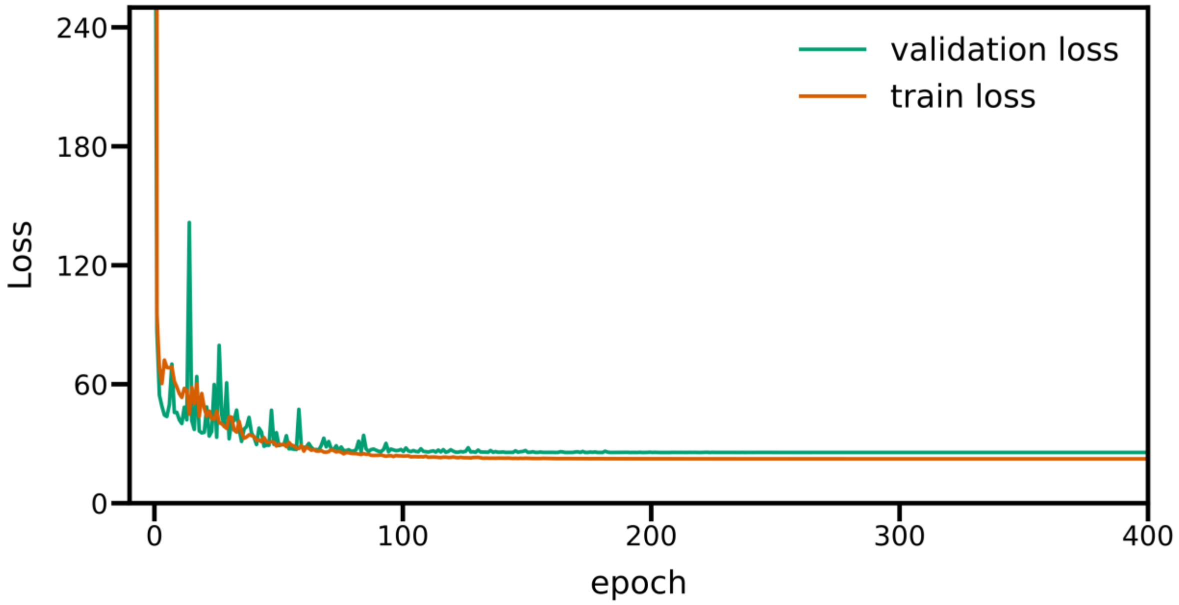

Supplementary Figure 1. The loss curves of the training and validation subsets in the final training with the optimized hyperparameters.

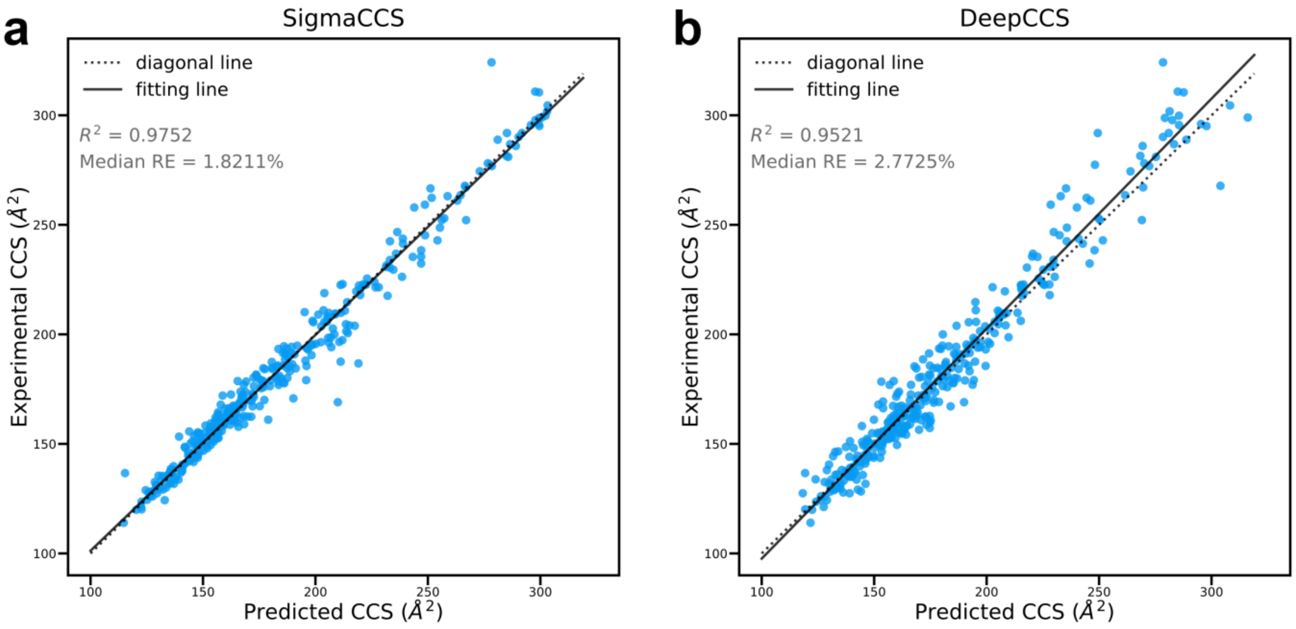

Supplementary Figure 2. Performance evaluation of SigmaCCS and DeepCCS on the external test set.

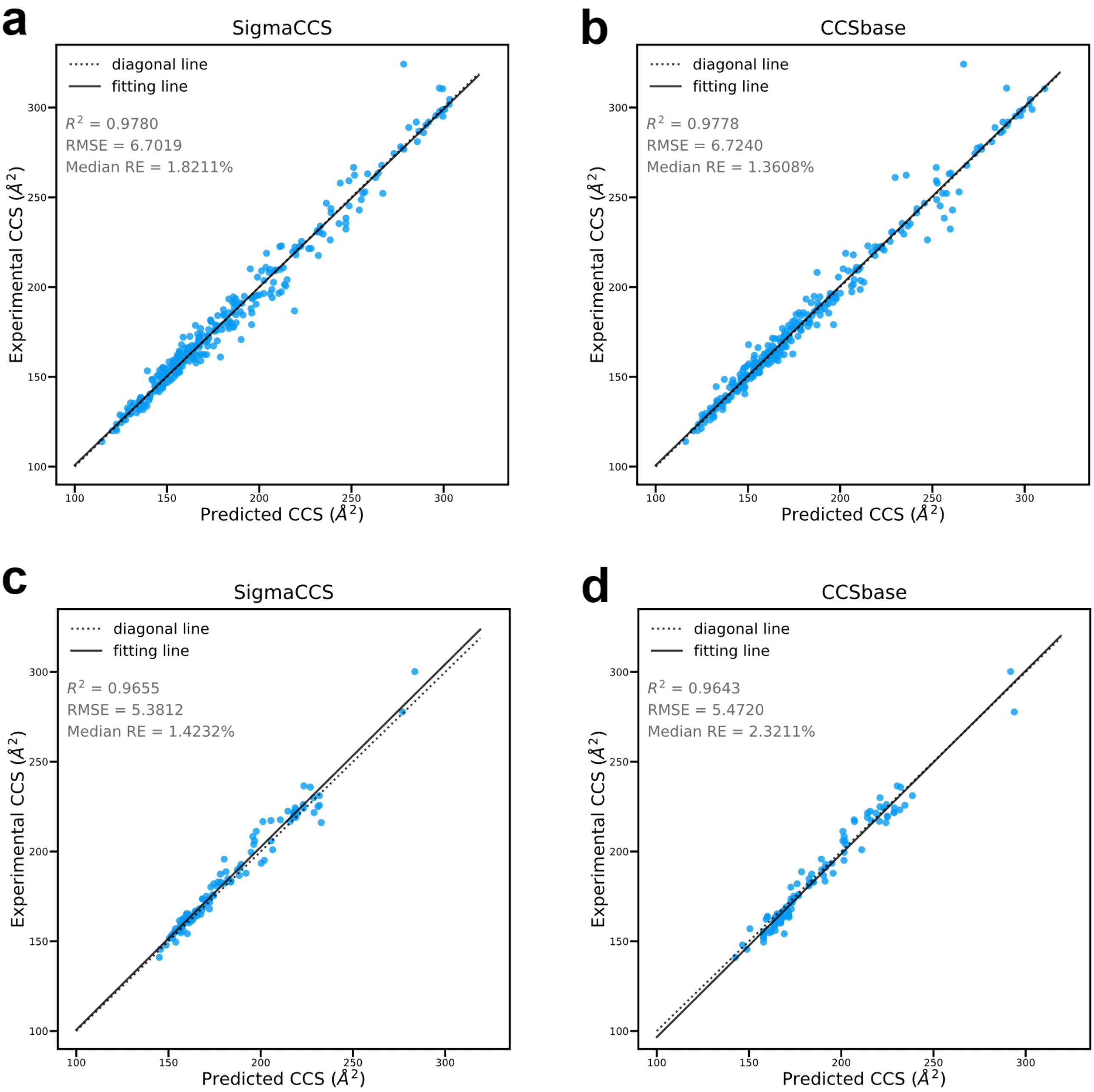

Supplementary Figure 3. Performance evaluation of SigmaCCS and CCSbase. a SigmaCCS on the external test set. **b** CCSbase on the external test set. **c** SigmaCCS on the plant dataset. **d** CCSbase on the plant dataset.

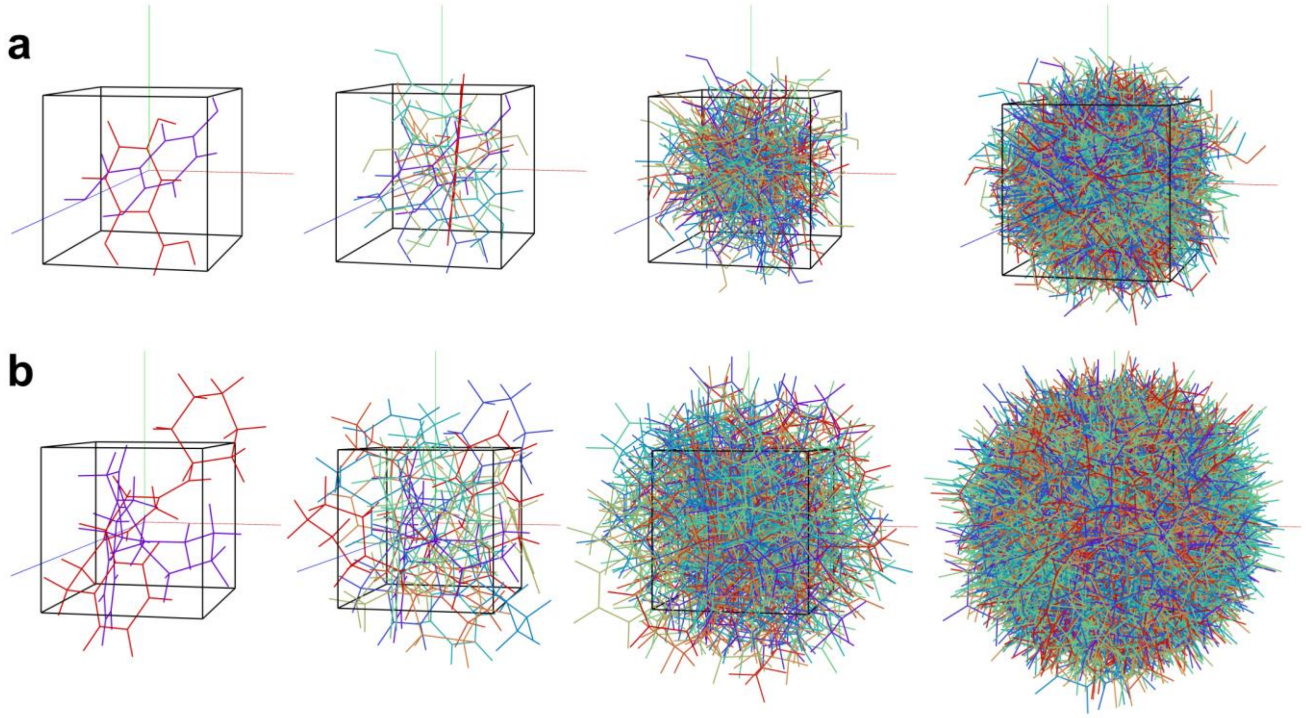

Supplementary Figure 4. Visualization of the 3D conformers of two randomly chosen molecules generated by ETKDG and MMFF94. Different numbers of conformers (2, 10, 100, and 1000) are shown from left to right, respectively. Different colors indicate different 3D conformers of the same molecule. **a** 2,5-dihydroxybenzoic acid. **b** Praziquantel.

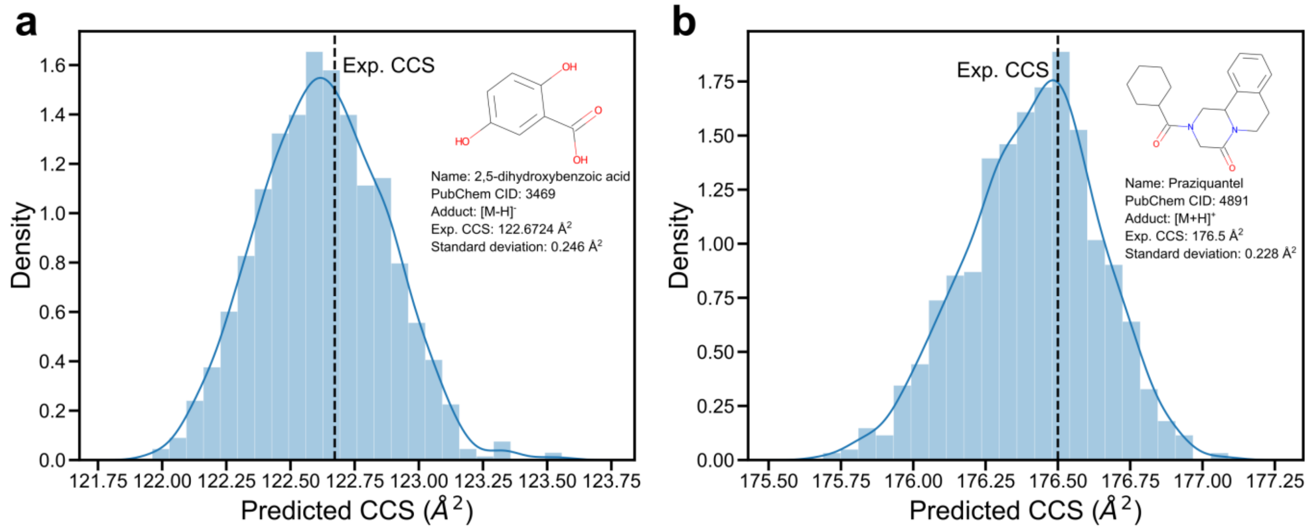

Supplementary Figure 5. Histograms with fitted density curves for the predicted CCS values of the molecules with 1000 different 3D coordinates generated by ETKDG and MMFF94. a 2,5-dihydroxybenzoic acid. **b** Praziquantel.

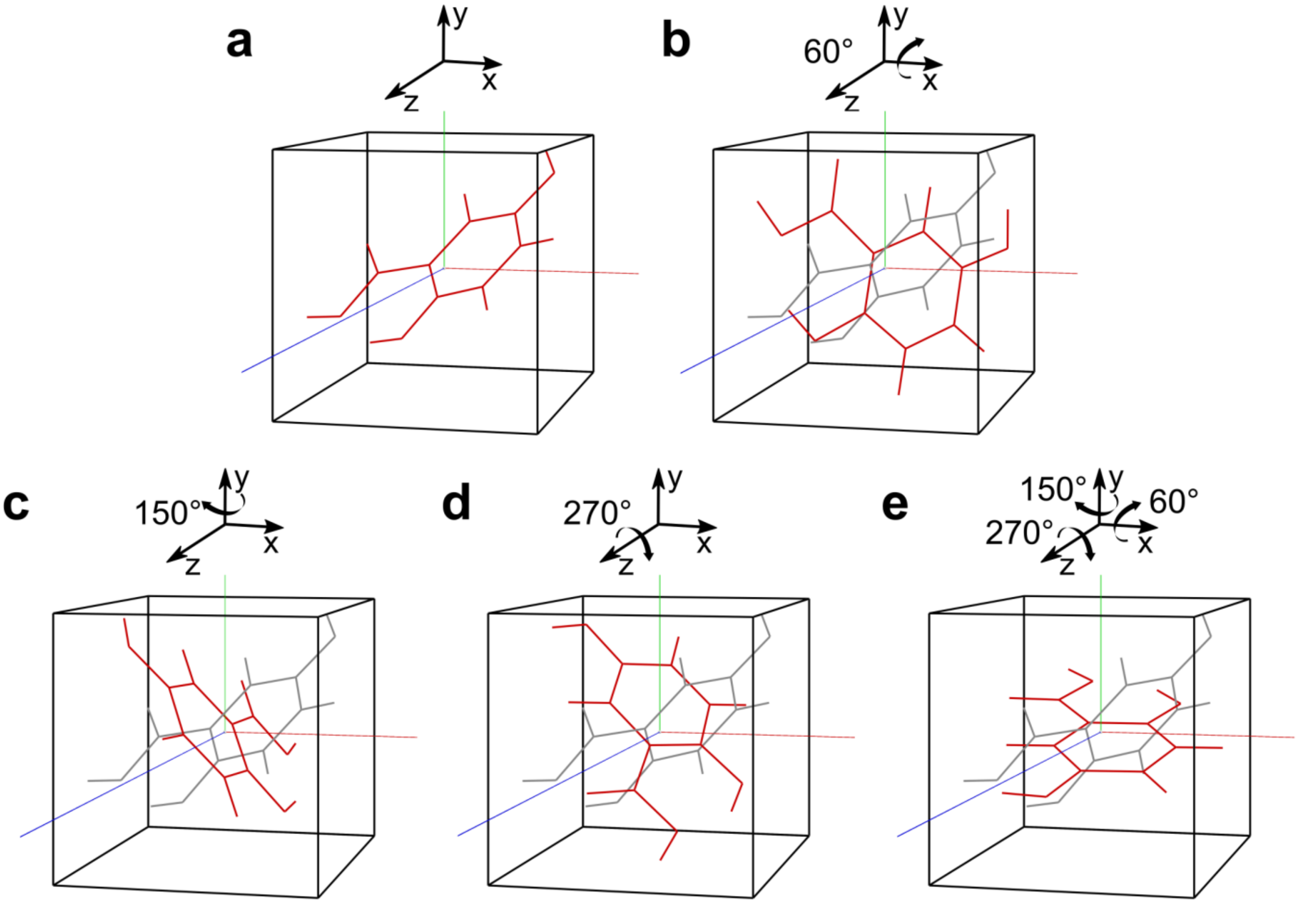

Supplementary Figure 6. Visualization of the 3D conformers of the molecule named 2,5-dihydroxybenzoic acid with completely random rotation. **a** Initial position of the 3D conformer. **b** The conformer rotates around the x-axis by a random rotation angle. **c** The conformer rotates around the y-axis by a random rotation angle. **d** The conformer rotates around the z-axis by a random rotation angle. **e** The conformer rotates around the x, y, and z-axes by random rotation angles. The grey conformer in **(b-e)** is the initial position before rotating the conformer. The rotation angles around the x, y, and z-axes are 60°, 150°, and 270°, respectively.

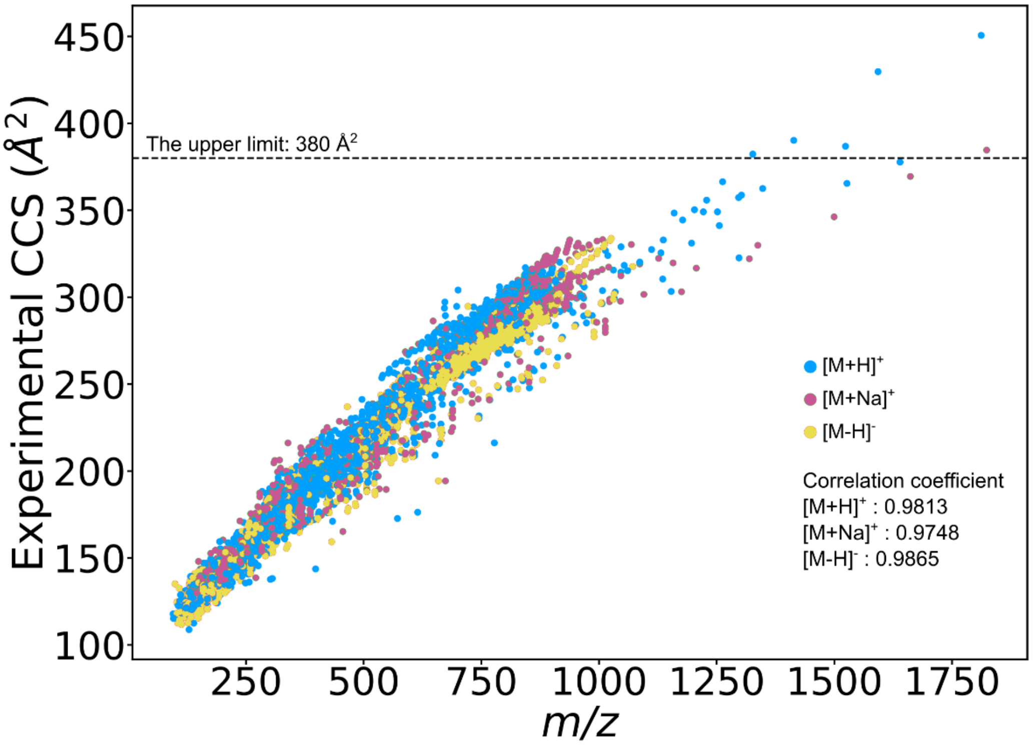

Supplementary Figure 7. Visual representation of experimental CCS *vs.* *m/z* for all adducts in the training and test sets of SigmaCCS.

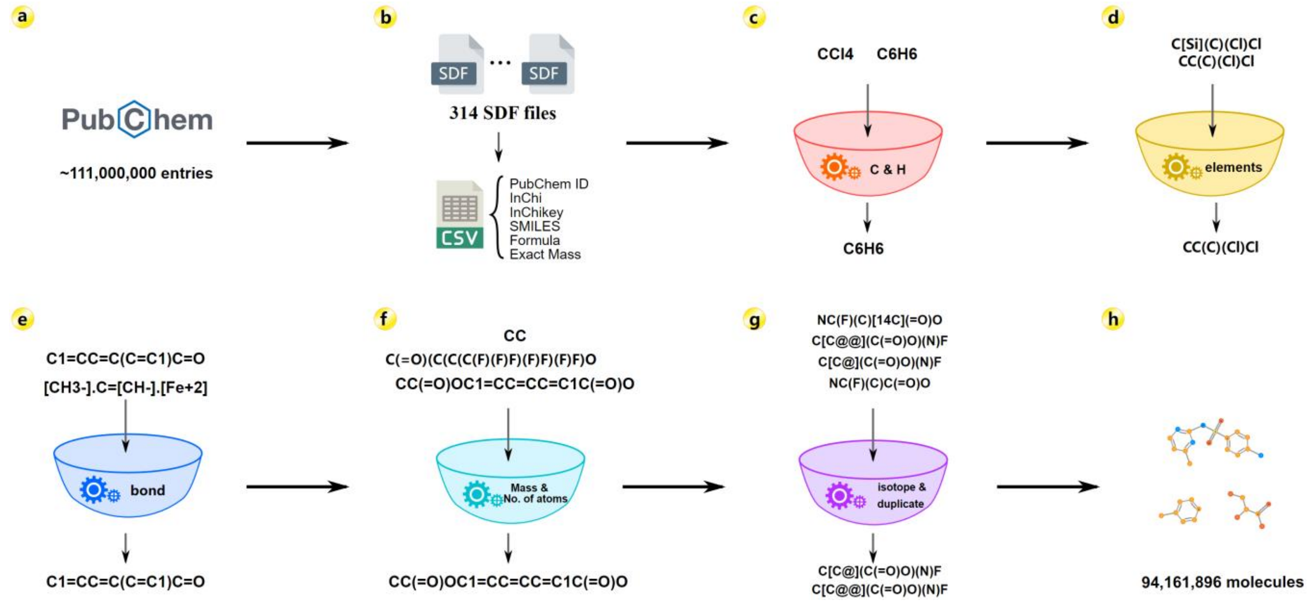

Supplementary Figure 8. Extraction and selection of proper molecules from PubChem to build the in-silico CCS database. a compound entries downloaded from the PubChem FTP site. **b** extraction of the PubChem ID, InChi, InChikey, SMILES, formula, and molecular weight from 314 structure data format (SDF) files. The extracted information was saved as the comma-separated values (CSV) files for further processing. **c** hydrogen and carbon filtering rule: Molecules without hydrogen or carbon were filtered out from the dataset. **d** elements filtering rule: The elements in the training set were C, H, O, N, P, S, F, Cl, Br, I, Co, As, and Se, and the molecules containing other elements were excluded from the dataset. **e** bond filtering rule: No molecules included ionic bonds in the training set. The molecules with "." in their SMILES strings were removed from the dataset. **f** molecular weight and atomic number filtering rule: the molecular weights of molecules should be between 100 and 1500. The atom number of F, Cl, Br, I, Co, As, and Se should not exceed 5. **g** isotopes and duplicate filtering rule: Molecules with isotopic in their SMILES strings were eliminated from the dataset since the training set of the SigmaCCS method did not contain this information. For some compounds, their chiral isomers and the Canonical SMILES are different PubChem IDs. We only kept their chiral isomers and removed the Canonical SMILES. **h** Remained 94,161,896 molecules after filtering the compounds in PubChem with the above rules. They were used for the subsequent CCS value prediction.

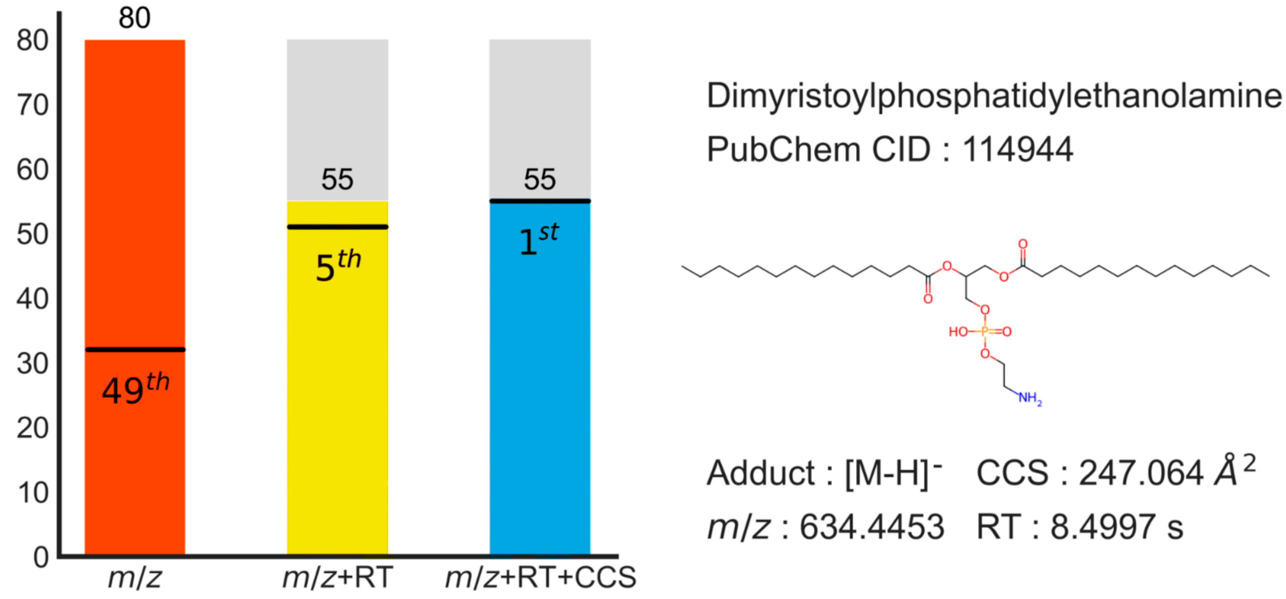

Supplementary Figure 9. Multidimensional filtering assisted by SigmaCCS. The ranking of the lipid (PubChem CID: 114944) by comparing the experimental data (*m/z*, RT, and CCS) with the theoretical or predicted data (molecular weight, RT predicted by GNN-RT, CCS predicted by SigmaCCS) of candidates.

Supplementary Table 1. Settings for some intuitive hyperparameters

Hyperparameter	Setting
Epoch	300
Batch size	14
Learning rate	0.0001
Optimizer	Adam
Activation function	ReLU
Regularizer	L2
Fully connected layers (Dense)	8
Number of nodes in fully connected layers (Dense)	384

Supplementary Table 2. Different combinations of two crucial hyperparameters

Type of graph layer	Number of layers	Layer1(in, out)	Layer2(in, out)	Layer3(in, out)	Pooling layers
ECC	3	ECC(23,16)	ECC(16,16)	ECC(16,16)	Global Sum Pool
ECC	2	ECC(23,16)	ECC(16,16)	—	
ECC	1	ECC(23,16)	—	—	
GCN	3	GCN(23,16)	GCN(16,16)	GCN(16,16)	
GCN	2	GCN(23,16)	GCN(16,16)	—	
GCN	1	GCN(23,16)	—	—	

Supplementary Table 3. The performance of the models with different hyperparameters on the validation subset

Model	Number of layers	R^2 (std)	Median RE% (std)
ECC	3	0.9933(0.0003)	1.327(0.1204)
ECC	2	0.9929(0.0009)	1.375(0.1498)
ECC	1	0.9917(0.0015)	1.581(0.1695)
GCN	3	0.9882(0.0035)	1.683(0.2872)
GCN	2	0.9864(0.0037)	1.962(0.3359)
GCN	1	0.9841(0.0081)	2.081(0.4482)

Supplementary Table 4. Evaluation of the randomness in parameter initialization on the performance of the models on the test set

No.	R^2	Median RE(%)
1	0.9937	1.297
2	0.9938	1.353
3	0.9940	1.235
4	0.9937	1.327
5	0.9941	1.258
6	0.9936	1.391
7	0.9940	1.214
8	0.9939	1.232
9	0.9942	1.163
10	0.9939	1.149
**Average(standard deviation)**	0.9939(0.0002)	1.262(0.0795)

Supplementary Table 5. Performance of SigmaCCS on the test set and the test set after molecular-level deduplication

**The test set after molecular-level deduplication**			**Test set**		
Size	R^2	Median RE (%)	Size	R^2	Median RE (%)
265	0.9930	1.241	559	0.9938	1.209
	0.9928	1.271		0.9938	1.199
	0.9933	1.276		0.9938	1.228
	0.9930	1.260		0.9938	1.246
	0.9931	1.205		0.9938	1.226
	0.9930	1.226		0.9938	1.188
	0.9929	1.218		0.9939	1.222
	0.9930	1.186		0.9939	1.228
	0.9931	1.156		0.9938	1.231
	0.9928	1.286		0.9938	1.254
Average	0.9930(0.00014)	1.232(0.0424)		0.9938(0.00004)	1.223(0.0202)

Supplementary Table 6. Comparison of SigmaCCS and DeepCCS on the test set

Test data size	R^2		Median RE (%)	
	SigmaCCS	DeepCCS	SigmaCCS	DeepCCS
	0.9940		1.194	
	0.9940		1.186	
	0.9941		1.205	
	**0.9945**		**1.175**	
514*	0.9943	0.9794	1.188	2.403
	0.9941		1.166	
	0.9941		1.239	
	0.9942		1.192	
	0.9943		1.150	
	0.9944		1.213	
Average	0.9942(0.00017)	0.9794	1.191(0.0249)	2.403

* The total number of molecules in the test set was 559. DeepCCS could not predict some molecules, so they were removed for fairness in the comparison. Only 514 molecules were retained. The model marked in bold is the chosen model for further applications.

Supplementary Table 7. Four molecules predicted by SigmaCCS resulted in the largest improvement compared to DeepCCS

PubChem CID	Molecules	SMILES	Adduct type	CCS value		
				Experiment	SigmaCCS	DeepCCS
6708694	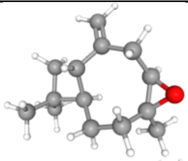	C=C1CC2OC2(C)CC[C@@H]2[C@@H]1CC2(C)C	$[M+H]^+$	144.2	144.8	157.3
5353532	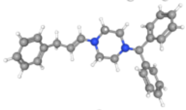	C(=C/N1CCN(C(c2ccccc2)c2ccccc2)CC1)\Cc1ccccc1	$[M+H]^+$	203.6	200.8	184.2
774	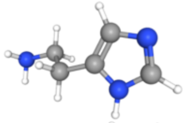	NCCc1cnc[nH]1	$[M+H]^+$	120.2	123.4	132.9
5359421	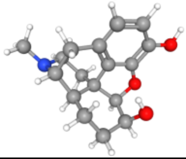	CN1CC[C@]23c4c5ccc(O)c4O[C@H]2[C@@H](O)CC[C@H]3[C@H]1C5	$[M+H]^+$	163.6	165.7	178.4

Supplementary Table 8. The 3 molecules with the largest relative error and the 3 molecules with the smallest relative error and their distances to the cluster centroids

PubChem CID	SMILES	Adduct	Relative error	Distances to the cluster centroids		
				cluster 1	cluster 2	cluster 3
390	C1=CN(C2(C(C(C(COP(O)(=O)OP(O)(=O)OC3(C(C(C(C(C(=O)O)(O3)[H])(O)[H])(O)[H])(O)[H])[H])(O2)[H])(O)[H])(O)[H])[H])C(N=C1O)=O	$[M+Na]^+$	0.00%	**5.5385**	8.6734	8.3617
993	C(C1(C(C(C(O)(O1)[H])(O)[H])(O)[H])[H])O	$[M+Na]^+$	0.00%	10.5819	6.7153	**3.8234**
86052	CC(C)CCCC(C)(CCCC(C)(CCCC1(C)CCC2=C(C)C(=CC(C)=C2O1)O)[H])[H]	$[M-H]^-$	0.02%	10.4298	5.9700	**3.5839**
10100278	CN1CCC2=CC(=C(C=C2C1CC3=CC=C(C=C3)OC)OC4=C(C=CC(=C4)CC5C6=CC(=C(C=C6CCN5C)OC)O)O)OC	$[M-H]^-$	11.78%	9.9133	9.8757	7.2097
5284447	[C@@]12(O[C@H]([C@@H]([C@H](C1)O)C(=O)O)C[C@@H](O[C@H]1[C@H]([C@H]([C@@H]([C@H](O1)C)O)N)O)/C=C/C=C/C=C/C=C/C[C@H](OC(=O)/C=C/[C@H]1O[C@@H]1C[C@@H](C2)O)C)O	$[M+H]^+$	11.97%	10.1498	12.6841	11.0748
16204181	C[C@H]1CC[C@]2([C@H]([C@H]3[C@@H](O2)C[C@@H]2[C@@]3(CC[C@H]3[C@H]2CC=C2[C@@]3(CC[C@@H](C2)O[C@H]2[C@@H]([C@H]([C@@H]([C@H](O2)CO)O[C@H]2[C@@H]([C@@H]([C@H]([C@@H](O2)C)O)O)O)O)O[C@H]2[C@@H]([C@@H]([C@H]([C@@H](O2)C)O)O)O)C)C)C)OC1	$[M-H]^-$	17.66%	9.2370	14.1969	12.6064

Supplementary Table 9. Performance of SigmaCCS on the test set with different coordinates generated by ETKDG and MMFF94

randomSeed	R^2	Median RE (%)	randomSeed	R^2	Median RE (%)
45	0.9938	1.204	66	0.9938	1.239
34	0.9938	1.203	65	0.9938	1.239
43	0.9938	1.278	90	0.9937	1.259
80	0.9936	1.231	60	0.9938	1.246
68	0.9936	1.220	32	0.9937	1.222
92	0.9939	1.183	67	0.9937	1.241
88	0.9938	1.212	49	0.9938	1.232
41	0.9938	1.247	46	0.9938	1.209
86	0.9937	1.222	13	0.9938	1.204
98	0.9937	1.255	36	0.9938	1.166
87	0.9937	1.218	69	0.9936	1.254
82	0.9937	1.272	52	0.9939	1.220
21	0.9938	1.255	37	0.9939	1.187
83	0.9938	1.230	35	0.9939	1.230
31	0.9939	1.206	91	0.9938	1.187
Average(standard deviation)		R^2 0.9938(0.00008)		Median RE 1.226(0.0269)	

Supplementary Table 10. Performance of SigmaCCS on the external test set with different coordinates generated by ETKDG and MMFF94

randomSeed	R^2	Median RE (%)	randomSeed	R^2	Median RE (%)
97	0.9793	1.871	79	0.9802	1.900
22	0.9793	1.881	12	0.9799	1.918
45	0.9798	1.892	49	0.9798	1.951
91	0.9795	1.891	2	0.9795	1.904
78	0.9799	1.871	21	0.9795	1.867
80	0.9796	1.954	33	0.9794	1.931
93	0.9797	1.954	16	0.9796	1.867
3	0.9799	1.878	7	0.9800	1.962
50	0.9793	2.006	69	0.9793	1.980
28	0.9792	1.969	14	0.9800	1.995
76	0.9802	1.871	60	0.9795	1.929
6	0.9797	1.976	87	0.9796	1.965
15	0.9793	2.007	13	0.9797	1.919
51	0.9796	1.939	65	0.9796	1.984
66	0.9797	1.928	62	0.9793	1.950
Average(standard deviation)		R^2 0.9796(0.00028)		Median RE 1.930(0.0445)	

Supplementary Table 11. Performance of SigmaCCS on the test set with completely random rotation angles

No.	R^2	Median RE(%)
Initial position	0.9938	1.209
completely random rotation	0.9937	1.183
completely random rotation	0.9938	1.207
completely random rotation	0.9938	1.165
completely random rotation	0.9937	1.234
completely random rotation	0.9938	1.197
completely random rotation	0.9937	1.243
completely random rotation	0.9938	1.213
completely random rotation	0.9938	1.226
completely random rotation	0.9938	1.180
completely random rotation	0.9937	1.206
completely random rotation	0.9938	1.238
completely random rotation	0.9939	1.237
completely random rotation	0.9938	1.233
completely random rotation	0.9937	1.239
completely random rotation	0.9938	1.214
completely random rotation	0.9938	1.223
completely random rotation	0.9938	1.207
completely random rotation	0.9937	1.220
completely random rotation	0.9939	1.219
Average(standard deviation)	0.9938(0.00006)	1.215(0.0213)

Supplementary Table 12. Results of the multidimensional lipids filtering

	m/z	*m/z* + RT	*m/z* + RT+ CCS
recall@1	15.2%	24.6%	28.9%
recall@10	24.8%	59.5%	63.5%
recall@20	34.3%	73.7%	79.6%
recall@30	47.6%	78.4%	91.2%
recall@40	58.3%	80.4%	94.7%

Supplementary Table 13. Source of experimental datasets

No.	CCS type	Data size	Content
1[12]	DT	498	Lipid, Peptide, Carbohydrate, Small Molecule
2[13]	DT	131	Lipid
3[14]	DT	847	Small Molecule
4[15]	DT	86	Peptide, Small Molecule
5[16]	DT	451	Lipid
6[17]	DT	949	Small Molecule
7[18]	DT	126	Peptide, Small molecule
8[19]	DT	1078	Small Molecule
9[20]	DT	405	Lipid
10[21]	DT	429	Lipid
11[22]	DT	336	Small Molecule
12[23]	TW	96	Small Molecule
13[24]	TW	257	Lipid
14[25]	TW	205	Small Molecule
15[26]	TW	1426	Peptide, Small Molecule
16[27]	TW	163	Lipid
17[28]	TW	357	Small Molecule
18[29]	TW	106	Small Molecule
19[30]	TW	173	Small Molecule
20[31]	TW	179	Lipid
21[8]	TIMS	2760	Lipid
22[32]	TIMS	2950	Lipid

Supplementary Table 14. Attributes of nodes (atoms), edges (chemical bonds), and ion types

Type	Attribute	Dimension
Node	One-hot encoding of the atom element	13
Node	One-hot encoding of the degree of the atom in the molecule, which is the number of directly-bonded neighbors (atoms)	5
Node	One-hot encoding of the atom radius	1
Node	Whether or not the atom is in a ring	2
Node	Atom mass	1
Node	Atom 3D coordinates	3
Edge	One-hot encoding of the bond type	4
Ion	One-hot encoding of the ion type	3

Supplementary Table 15. The atomic mass and radius used to construct molecular graphs

Atom type	Atomic covalent radii(pm)	Atomic weights
H	32	1.00794
C	75	12.0107
N	71	14.0067
O	63	15.9994
F	64	18.9984032
P	111	30.973762
S	103	32.065
Cl	99	35.453
Co	111	58.933195
As	121	74.92160
Se	116	78.96
Br	114	79.904
I	133	126.90447

REFERENCES

1. Ross DH, Cho JH, Xu LB. Breaking Down Structural Diversity for Comprehensive Prediction of Ion-Neutral Collision Cross Sections. *Analytical Chemistry* **92**, 4548-4557 (2020).
2. Schroeder M, Meyer SW, Heyman HM, Barsch A, Sumner LW. Generation of a Collision Cross Section Library for Multi-Dimensional Plant Metabolomics Using UHPLC-Trapped Ion Mobility-MS/MS. *Metabolites* **10**, (2020).
3. Paglia G, Smith AJ, Astarita G. Ion mobility mass spectrometry in the omics era: Challenges and opportunities for metabolomics and lipidomics. *Mass Spectrometry Reviews* **41**, 722-765 (2022).
4. Blaney JM, Dixon JS. Distance geometry in molecular modeling. *Reviews in computational chemistry*, 299-335 (1994).
5. Wang YL, Xiao JW, Suzek TO, Zhang J, Wang JY, Bryant SH. PubChem: a public information system for analyzing bioactivities of small molecules. *Nucleic Acids Research* **37**, W623-W633 (2009).
6. Kim S*, et al.* PubChem Substance and Compound databases. *Nucleic Acids Research* **44**, D1202-D1213 (2016).
7. Kim S*, et al.* PubChem 2019 update: improved access to chemical data. *Nucleic acids research* **47**, D1102-D1109 (2019).
8. Tsugawa H*, et al.* A lipidome atlas in MS-DIAL 4. *Nature Biotechnology* **38**, 1159-+ (2020).
9. Yang Q, Ji HC, Lu HM, Zhang ZM. Prediction of Liquid Chromatographic Retention Time with Graph Neural Networks to Assist in Small Molecule Identification. *Analytical Chemistry* **93**, 2200-2206 (2021).
10. Yang Q, Ji HC, Fan XQ, Zhang ZM, Lu HM. Retention time prediction in hydrophilic interaction liquid chromatography with graph neural network and transfer learning. *Journal of Chromatography A* **1656**, (2021).
11. Domingo-Almenara X*, et al.* The METLIN small molecule dataset for machine learning-based retention time prediction. *Nature Communications* **10**, (2019).
12. May JC*, et al.* Conformational Ordering of Biomolecules in the Gas Phase: Nitrogen Collision Cross Sections Measured on a Prototype High Resolution Drift Tube Ion Mobility-Mass Spectrometer. *Analytical Chemistry* **86**, 2107-2116 (2014).
13. Paglia G*, et al.* Ion Mobility Derived Collision Cross Sections to Support Metabolomics Applications. *Analytical Chemistry* **86**, 3985-3993 (2014).
14. Groessl M, Graf S, Knochenmuss R. High resolution ion mobility-mass spectrometry for separation and identification of isomeric lipids. *Analyst* **14**, 6904-6911 (2015).
15. Zhou ZW, Shen XT, Tu J, Zhu ZJ. Large-Scale Prediction of Collision Cross-Section Values for Metabolites in Ion Mobility-Mass Spectrometry. *Analytical Chemistry* **88**, 11084-11091 (2016).
16. Hines KM, Herron J, Xu LB. Assessment of altered lipid homeostasis by HILIC-ion mobility-mass spectrometry-based lipidomics. *Journal of Lipid Research* **58**, 809-819 (2017).
17. Bijlsma L*, et al.* Prediction of Collision Cross-Section Values for Small Molecules: Application to Pesticide Residue Analysis. *Analytical Chemistry* **89**, 6583-6589 (2017).
18. Hines KM, Ross DH, Davidson KL, Bush MF, Xu LB. Large-Scale Structural Characterization of Drug and Drug-Like Compounds by High-Throughput Ion Mobility-Mass Spectrometry. *Analytical Chemistry* **89**, 9023-9030 (2017).
19. Stow SM*, et al.* An Interlaboratory Evaluation of Drift Tube Ion Mobility-Mass Spectrometry Collision Cross Section Measurements. *Analytical Chemistry* **89**, 9048-9055 (2017).
20. Zhou ZW, Tu J, Xiong X, Shen XT, Zhu ZJ. LipidCCS: Prediction of Collision Cross-Section Values for Lipids with High Precision To Support Ion Mobility-Mass Spectrometry-Based Lipidomics. *Analytical Chemistry* **89**, 9559-9566 (2017).
21. Zheng XY*, et al.* A structural examination and collision cross section database for over 500 metabolites and xenobiotics using drift tube ion mobility spectrometry. *Chemical Science* **8**, 7724-7736 (2017).
22. Hines KM*, et al.* Characterization of the Mechanisms of Daptomycin Resistance among Gram-Positive Bacterial Pathogens by Multidimensional Lipidomics. *Msphere* **2**, (2017).
23. Lian R*, et al.* Ion mobility derived collision cross section as an additional measure to support the rapid analysis of abused drugs and toxic compounds using electrospray ion mobility time-of-flight mass spectrometry. *Analytical Methods* **10**, 749-756 (2018).
24. Mollerup CB, Mardal M, Dalsgaard PW, Linnet K, Barron LP. Prediction of collision cross section and retention time for broad scope screening in gradient reversed-phase liquid chromatography-ion mobility-high resolution accurate mass spectrometry. *Journal of Chromatography A* **1542**, 82-88 (2018).
25. Righetti L, Bergmann A, Galaverna G, Rolfsson O, Paglia G, Dall'Asta C. Ion mobility-derived collision cross section database: Application to mycotoxin analysis. *Analytica Chimica Acta* **1014**, 50-57 (2018).
26. Tejada-Casado C*, et al.* Collision cross section (CCS) as a complementary parameter to characterize human and veterinary drugs. *Analytica Chimica Acta* **1043**, 52-63 (2018).
27. Nichols CM*, et al.* Untargeted Molecular Discovery in Primary Metabolism: Collision Cross Section as a Molecular Descriptor in Ion Mobility-Mass Spectrometry. *Analytical Chemistry* **90**, 14484-14492 (2018).
28. Hines KM, Xu LB. Lipidomic consequences of phospholipid synthesis defects in Escherichia coil revealed by HILIC-ion mobility-mass spectrometry. *Chemistry and Physics of Lipids* **219**, 15-22 (2019).
29. Leaptrot KL, May JC, Dodds JN, McLean JA. Ion mobility conformational lipid atlas for high confidence lipidomics. *Nature Communications* **10**, (2019).
30. Blazenovic I*, et al.* Increasing Compound Identification Rates in Untargeted Lipidomics Research with Liquid Chromatography Drift Time-Ion Mobility Mass Spectrometry. *Analytical Chemistry* **90**, 10758-10764 (2018).
31. Vasilopoulou CG*, et al.* Trapped ion mobility spectrometry and PASEF enable in-depth lipidomics from minimal sample amounts. *Nature Communications* **11**, (2020).
32. Poland JC, Leaptrot KL, Sherrod SD, Flynn CR, McLean JA. Collision Cross Section Conformational Analyses of Bile Acids via Ion Mobility-Mass Spectrometry. *Journal of the American Society for Mass Spectrometry* **31**, 1625-1631 (2020).
