# Supplementary material for: Highly accurate and large-scale collision cross sections prediction with graph neural networks
Source: Commun Chem. 2023 Jul 4;6:139. doi: 10.1038/s42004-023-00939-w (PMC10319785; doi:10.1038/s42004-023-00939-w)
Supplement: Supplementary file 2 — Description of Additional Supplementary Files [file 42004_2023_939_MOESM2_ESM.pdf]

Description of Additional Supplementary Files

File name: Supplementary Data 1

Description: The curated dataset for training, validating, and testing the SigmaCCS model.

File name: Supplementary Data 2

Description: Molecules in CCSbase and PubChem whose 3D conformers can not be generated or optimized by ETKDG and MMFF94.

File name: Supplementary Data 3

Description: Forty-three outliers detected in the curated dataset.

File name: Supplementary Data 4

Description: Chemical classification of the molecules in the curated dataset using the ClassyFire web service.

File name: Supplementary Data 5

Description: The external test set curated from the dataset of AllCCS by deduplicating molecules in the training set of SigmaCCS.

File name: Supplementary Data 6

Description: The plant dataset by deduplicating molecules in the training set of SigmaCCS.
